# Detailed global modelling of soil organic carbon in cropland, grassland and forest soils

**DOI:** 10.1371/journal.pone.0222604

**Published:** 2019-09-19

**Authors:** Tiago G. Morais, Ricardo F.M. Teixeira, Tiago Domingos

**Affiliations:** MARETEC–Marine, Environment and Technology Centre, LARSyS, Instituto Superior Técnico, Universidade de Lisboa, Lisbon, Portugal; Oak Ridge National Laboratory, UNITED STATES

## Abstract

Assessments of the global carbon (C) cycle typically rely on simplified models which consider large areas as homogeneous in terms of the response of soils to land use or consider very broad land classes. For example, “cropland” is typically modelled as an aggregation of distinct practices and individual crops over large regions. Here, we use the process-based Rothamsted soil Carbon Model (RothC model), which has a history of being successfully applied at a global scale, to calculate attainable SOC stocks and C mineralization rates for each of c. 17,000 regions (combination of soil type and texture, climate type, initial land use and country) in the World, under near-past climate conditions. We considered 28 individual crops and, for each, multiple production practices, plus 16 forest types and 1 grassland class (total of 80 classes). We find that conversion to cropland can result in SOC increases, particularly when the soil remains covered with crop residues (an average gain of 12 t C/ha) or using irrigation (4 t C/ha), which are mutually reinforcing effects. Attainable SOC stocks vary significantly depending on the land use class, particularly for cropland. Common aggregations in global modelling of a single agricultural class would be inaccurate representations of these results. Attainable SOC stocks obtained here were compared to long-term experiment data and are well aligned with the literature. Our results provide a regional and detailed understanding of C sequestration that will also enable better greenhouse gas reporting at national level as alternatives to IPCC tier 2 defaults.

## Introduction

Understanding terrestrial carbon (C) cycle dynamics is essential to assess greenhouse gas (GHG) emissions and to mitigate and adapt to climate change [1–3]. Land use and land use change (LU/LUC) are the second most relevant anthropogenic source of C into the atmosphere, after emissions from fossil fuel combustion [4]. Long-term transformations from forest and grassland to cropland are known to deplete soil organic carbon (SOC) stocks [5–7] releasing C into the atmosphere. SOC can be replenished through C sequestration [8,9]. SOC is strongly linked with soil management practices (e.g. mulching), soil proprieties (e.g. texture), climate (e.g. temperature and rainfall) [8,10,11]. These factors display high spatial variability [9,10,12] and make terrestrial C fluxes the most uncertain in the global C cycle [13]. Besides temporal and spatial variability, SOC measurement is also significantly uncertain. Even in unperturbed systems, this means that changes in the soil C pool can take 5 or more years to be detectable [14,15]. Linking SOC and LU is additionally difficult as global LU maps and databases typically have a low number of classes or lack relevant information [16]. The European Land Use/Land Cover Area Frame Survey (LUCAS) [17] database jointly reports SOC and LU class for c. 19,000 field measurements that will continue to be repeated into the future. However, LUCAS does not include any information regarding how long after the last LUC event the measurement was taken. The dynamics of SOC after LUC and the legacy effects of prior LU are relevant for the determination of potential SOC stocks and how fast SOC is mineralized [18].

In 2006, the Intergovernmental Panel on Climate Change (IPCC) proposed a method [19] with three tiers of detail to account for changes in soil C stocks due to LU/LUC. This method has been applied at local [20], regional [21,22] and global scales [23]. Tier 1 has default factors of emissions and sequestration of LU/LUC. Tier 2 can potentially incorporate country-specific management systems and biophysical data to calculate SOC stocks for mineral and organic soils. Emission/sequestration C flows between atmosphere and soil are then calculated as the yearly change in SOC stocks over 20 years. Nevertheless, Tier 2 approaches still rely heavily on default factors for unspecific LU classes within major LU types (cropland, forest, pasture) [19], as shown in the inventories to the United Nations Framework Convention on Climate Change and submitted to the Kyoto Protocol by countries such as Canada [24], France [25] and Portugal [26]. Consequently, comparisons of the IPCC method with regionalized models have revealed large discrepancies regarding SOC changes [20,21].

The alternative for global and regional large-scale assessments of SOC change is to use process-based models. Process-based soil models consider biogeochemical processes formulated according to mathematical-ecological theory. They are capable of simulating SOC turnover according to specific site conditions and relating it to management practices [27–29]. They address user-defined temporal and spatial scales based on scenarios that characterize intra and inter-annual dynamics. The application of these models transfers the need for highly regionalized data from the output variable (i.e. SOC) to the input variables. The Rothamsted Carbon Model (RothC) [30] model is one of the most commonly used soil models today [30–35]. The set of inputs required by RothC is a crucial advantage when compared with other process-based soil models that require much larger datasets [33]. The range of dynamic SOC processes captured by RothC is lower, but the fact that it is a parsimonious model makes RothC a good candidate for global modelling exercises due to its manageability. However, few examples exist of continental or global-scale applications of RothC [32,36], which had either low spatial resolution or a low number of classes. For example, Gottschalk et al. [32] applied it globally to gain insight on future SOC stocks under scenarios of LU distribution, but including only three LU classes.

As the past few years have seen a surge in computational power and in global data availability for modelling [29], here we propose new Tier 2-equivalent defaults obtained from global SOC modelling with a modified version of RothC. The main innovative features of the modelled data obtained here are (a) the level of spatial differentiation of the results (and respective uncertainty), and (b) the number of land classes, particularly crop types. Regarding (a), the basic unit of analysis were around 17,000 unique homogeneous territorial units (UHTU), which result from the spatial combination of thermal zones, land cover, soil type, soil texture and countries. Regarding (b), we calculated global SOC dynamics for 80 specific LUs within broad LU classes (croplands, forests and grassland). The main results targeted with this study were in particular a new set of highly spatially differentiated and land class-specific attainable SOC stocks and C mineralization rates. Attainable SOC is the maximum attainable SOC with occupation by a given vegetation type and climate conditions [37]. We calculate it as a present potential due to the fact that we assume constant near-past climate and soil conditions, as well as current crop yields, in its determination.

## Materials and methods

### The RothC model

The Rothamsted Carbon Model version 26.3 (RothC) is a model of C turnover in non-waterlogged soils [30]. It was initially developed to model C turnover in arable soils and later expanded to grasslands and forests [3,30,31]. It takes into account the effects of temperature, moisture content and soil type. The model uses a monthly step. SOC is divided in five compartments or pools, depending on decomposability: inert organic matter (IOM), easily decomposable plant material (DPM), resistant plant material (RPM), microbial biomass (BIO) and humified organic matter (HUM). The IOM pool is resistant to decomposition and does not receive C inputs [38]. Each compartment except IOM decomposes according to a first-order decomposition process. RothC has been applied using data from long-term experiments across several ecosystems, climate conditions, and land use (LU) classes [3,30,31].

### Calculation procedure

**Fig 1:**
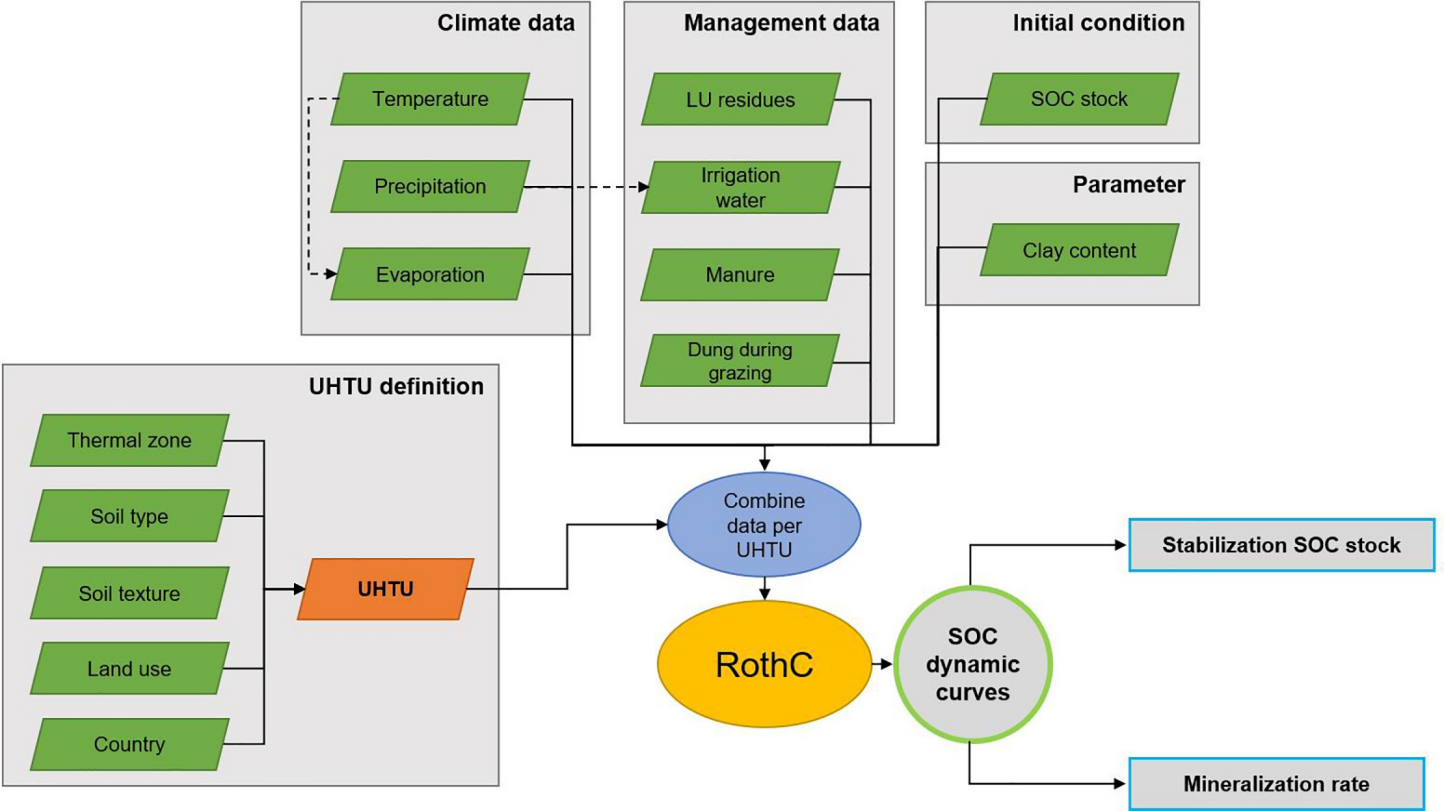
Workflow procedure for data preparation to be implemented in the RothC model. LU–Land use; SOC–Soil organic carbon; UHTU—Unique Homogeneous Territorial Unit.

The calculation procedure is depicted in Fig 1 and summarized as follows. The details for each of these steps are presented in the ensuing sub-sections. First, we collected the source data for all input variables presented in Fig 1 (i.e. climate and management data, initial SOC stock and clay content) and respective uncertainty, assuming normal distributions. These data involve all climate variables (temperature, precipitation and evaporation), management variables (water input, residues production per LU and country as function of the yield, farmyard manure and organic fertilizer application per LU) and initial SOC stock and clay content. Then we defined c. 17,000 Unique Homogeneous Territorial Units (UHTUs) that are assumed to be similar in terms of soil type, climate regime and current LU. We assigned all input variables to each UHTU. We then selected priority LU classes and a set of management practices for cropland production. In each UHTU only a subset of the list of LU classes is biophysically feasible. The next step was to insert all variables into a MATLAB version (produced in this paper) of RothC. We ran model initialization to divide the initial SOC stock under current land use into the five C pools using the method proposed by Weihermüller et al. [39]. Then, we used RothC one hundred times in each UHTU for each feasible LU class and for 86 years, in order to simulate results that take into consideration intra-UHTU variability and obtain uncertainty measures for average UHTU-level results. Results were then used to fit a two-parameter saturating exponential model for each UHTU. We calculated attainable SOC stocks and mineralization rates per LU class and UHTU using those two parameters. Finally, we compared results to long-term SOC measurements and data collected in international projects for a wide range of geographical regions.

#### Input data

Fig 1 indicates the land, soil and climate data inputs required by RothC [30]. Crop parameters involved in calculations are soil cover period, monthly input of plant residues (t C/ha), farmyard manure (stored and sprayed manure/slurry and direct deposition of excretions during grazing) C inputs (t C/ha) and irrigation (mm). The clay content of soils (%) is used to determine the initial distribution of SOC between pools. The starting SOC content (t C/ha) is used to initialize the model and also the CO_2_ flux from the soil in the first simulation period. As for climate data, the model requires average monthly mean air temperature (°C), precipitation (mm) and open pan evaporation (mm). We considered a “static world” scenario, i.e. there was no change in climate in the future. This provides potential SOC dynamics in near-past conditions but cannot be extrapolated for forecasting the role of climate change in SOC. We assigned uncertainty to all input data using mean values and standard deviations. We assumed a normal probability distribution for all parameters.

Soil cover period is required due to higher decomposability of C in uncovered soils [30]. This is a binary monthly variable, where 1 means that the soil was covered with vegetation during that month and 0 means that the soil was uncovered. Soil cover period was set to 1 for permanent crops (orchards, olive groves and vineyards), forests, grasslands and shrublands. For croplands, this parameter was obtained from Chapagain et al. [40] and depends on the thermal zones used by the Global Agro-ecological Zones (GAEZ) Project [41], whose definition is based on temperature and precipitation. They provide sowing/planting dates and the duration of the vegetation cycles. In the months between sowing/plantation and harvesting we considered the soil cover parameter to be 1 and in the other months, in the case where residues are not left on the field and so the soil is bare, we assigned it the value 0. For cereals, when residues are left on the field we used the value 1 for all months.

To estimate annual plant residue C input, we used the IPCC methods [19,42,43]. Permanent and annual crop residues are equal to a fraction of above-ground crop yield plus the below-ground residues [30]. Below-ground residues are also a fixed fraction of the above-ground yield. Yield data at country-level was obtained from the Food and Agriculture Organization of the United Nations (FAO) [44]. We used the average and standard deviation of yields between 2004 and 2014. For forests and grasslands, residue inputs are the biomass left to decay on the ground (e.g. litter), which depends on the vegetation type and climate region. The full method description and data used for crop and forest and grassland is in S1 File. The method used for croplands determines C residues for an entire year. We then distributed the residues monthly. This distribution depends on the distribution of net primary production per month and the life stages of plants. We used the method proposed by Jebary et al. and Morais et al. [33,45,46] to distribute residue C input to soil between months. According to this method, for cereal crops, 50% of residues were allocated to the harvesting month and the remaining 50% were equally distributed among the three months before. For permanent crops, 70% of residues were allocated to the pruning months and the remaining 30% to the prior four months. This distribution depends on the monthly distribution of net primary production and life stages of plants. The months for harvesting and pruning were obtained from Chapagain et al. [40]. For forests and grasslands, residues were equally distributed during the year, thus assuming that there are no management activities.

Farmyard manure is an extra C input in croplands and grasslands. In grasslands, the source of this input is both direct dung deposition during grazing and (possibly) application of manure, while in croplands only the latter is applicable. For dung excretions during grazing we used livestock units on grasslands from Gridded Livestock of the World v2.0 [47] combined with manure excretion from IPCC [19] for cattle, sheep and goat livestock. For manure application in grass and croplands there is no known data available. Organic fertilization is highly variable, as it depends on local manure availability and price, its source and type (solid, liquid) and its chemical composition (humidity, C:N ratio, etc.). We used data from Mueller et al. [48] for total aggregate consumption of nitrogen (N) fertilizers from synthetic and animal origin, and considered three scenarios. In the first scenario, the total consumption of N from Mueller et al. [48] was assumed to be synthetic fertilizer only, and thus there is no manure input. In the second, we considered that only 50% of the total amount of fertilizer was applied as manure. In the third scenario, we considered that all N used as fertilizer was manure (organic fertilizer). To convert the N content of manure to C, we used the C:N ratio of farmyard manure provided by FAO [49]. We excluded any vegetable material applied as part of manure (such as straw and other materials from animal beddings) to avoid double-counting, as plant-based C input is already included in cases where residues are left on the field. The detailed method is in S1 File.

RothC does not distinguish between water input types (i.e. water from irrigation and water from precipitation). Water provision during dry seasons can make a difference in SOC mineralization [38]. We used the method of Pfister et al. [50] to calculate water input as the sum of precipitation and irrigation. The method assumes that, for each crop and attainable yield, the water requirements of that crop must be attained through precipitation alone or with additional irrigation. Irrigation is either zero (if precipitation is higher than the water needed for a given month) or the difference between the water needed by the crop and the precipitation for each month. Crop water requirements were obtained by multiplying specific crop coefficients depending on a monthly growth stage factor and potential evapotranspiration. The growth stage factors were obtained from Chapagain et al. [40] for all crops in each thermal zone. Potential evapotranspiration was obtained using the Thornthwaite equation [51], which uses monthly average air temperature, average day length, in hours, and number of days per month obtained from MODIS [52]. The detailed method description and data used for croplands is in S1 File.

Average monthly precipitation and mean air temperature were both obtained from National Aeronautics and Space Administration’s (NASA). Precipitation was obtained from the database of the “Global Precipitation Climatology Project (GPCP)” [53] We used data for thirteen years (2000–2013). To establish normal climatic conditions, 30 years are usually necessary. However, the data from NASA does not go so far back. Additionally, given recent changes in climate that produce many anomalous meteorological years, we believe a shorter period can nevertheless more accurately represent near-past conditions [54–56]. Further, we also used this period to ensure consistency with other data, as yield data is also valid for the same period only. Monthly open pan evaporation was calculated as 75% of the potential evaporation, an assumption used by previous studies using RothC [33,46]. Potential evaporation was calculated using the Thornthwaite equation [51].

SOC initialization values were obtained from the European Soil Data Centre (ESDAC) [57]. Clay content was obtained from the Harmonized World Soil Database [58]. The topsoil layer considered was 30 cm deep.

#### Definition of unique homogeneous territorial units

In this study, we used the concept of UHTU to establish the basic units of analysis. UHTUs are geographical regions where soil type and texture, climate type and LU are uniform at the scale of analysis. RothC was used for each unique region individually. UHTUs were defined as a geographical combination of five layers, namely thermal zones, land cover, soil type, soil texture and country. Thermal zones were obtained from GAEZ [41], which divides the world into 12 zones (S1 File). We excluded arctic and desert regions. We used data from the Land Processes Distributed Active Archive Center (LP DAAC) [59] for attribution of land classes. This source considers 16 LU classes, out of which we excluded “water” and “unclassified” regions from the analysis. Soil type and texture data were obtained from the World Reference Base (WRB), as depicted in Fischer et al. [60]. We considered the 8 types of soil and 13 types of texture from WRB. Finally, it was necessary to use a country layer in the definition of UHTUs because crop yield data from FAO is only available at country-level. We obtained the country borders from the World Borders Dataset [61]. We used FAO yield data for 202 countries and for the period 2004–2014. The full list of countries is in S1 File. The combination of these data sets resulted in 17,203 UHTUs (S3 File). An individual set of RothC simulations was then carried out for each UHTU. The average area of each UHTU is 6,400 km2.

#### Choice of land use classes

We performed simulations of SOC dynamics for 43 LU classes, including 28 agricultural classes (under different management practices), 16 forest classes and 1 grassland class. The agricultural classes selected were the 28 most produced and traded (which are the result of intersecting the top twenty most produced and traded products) during the period 2004–2014 (most recent year available) [44] in the World (using FAOSTAT nomenclature): apples; bananas; barley; cabbages and other brassicas; carrots and turnips; cocoa, beans; coconuts; coffee, green; grapes; groundnuts, with shell; maize; oil, palm fruit; olives; onions, shallots, green; oranges; potatoes; rapeseed; rice, paddy; seed cotton; sorghum; soybeans; sugar beet; sugar cane; sunflower seed; sweet potatoes; tobacco, unmanufactured; tomatoes; and wheat. Regarding management practices, besides the three organic fertilization scenarios explained previously, we considered rainfed and irrigated production (except cabbage, carrot and onion, in which cases we only used irrigated production because rainfed areas are insignificant compared with irrigated areas). We considered two management options for residue management of cereals: (1) residues are left on the field; (2) residues are removed from the field. Forest and grassland classes were obtained from the IPCC classification [19,43]: broadleaf deciduous forest and needleleaf evergreen forest in the dry boreal zone, moist boreal zone, moist cold temperate zone, dry cold temperate zone, dry warm temperate zone, moist warm temperate zone, sub-tropical zone and tropical zone. The IPCC method [43] for forest residues provides annual C residues and transition time for all forests. Thus, we used the linear annual litter increase rate during forest growth and after maturity a constant annual C residue input. IPCC considers only one type of grassland. In total there are 80 possible combinations, combining cropland with management practices (irrigation/rainfed and residues left/removed on/from the field) plus the forest classes and the grassland class. To determine the UHTUs where each agricultural LU class is feasible, country-level FAOSTAT yield data is insufficient. Instead, we used production capacity maps from GAEZ [41]. These maps integrate climatic and edaphic variables to determine feasible areas for each agricultural LU class. For each crop, if there is at least one pixel inside of the UHTU with non-null production capacity in the present, we assumed that the crop is feasible in that UHTU. Forest classes are divided by thermal zone by definition. For grasslands, we used directly the areas classified as “grassland” from LP DAAC maps [59].

#### MATLAB implementation

RothC can be obtained under a free for research license from the Rothamsted Research website [62]. However, that version of the model only runs for one region (UHTU) and one LU class at a time. To expedite calculations for all UHTUs simultaneously we implemented a Matlab version of RothC. We implemented also a Monte Carlo approach [63] for determining the uncertainty of results. We ran the model 100 times for each LU class in each UHTU. Each iteration thus used a unique set of input parameters drawn randomly from their respective normal probability distributions.

#### Calculation of attainable SOC stocks and mineralization rates

We obtained the annual SOC stock from the model for 86 years (simulation between 2014 and 2100). As 2000–2013 climate data was used, this estimation considers climate stability and does not take the role of climate change into account.

RothC is a multiple pool model but the overall shape of the dynamic SOC curves for each pool is approximately exponential. For grassland and agricultural classes, this approximation is a good fit for all periods simulated because the C input from residues is constant, but for forest classes it is only true after maturity (i.e. when annual C residues are constant). In the transition period, dynamic SOC curves are approximately a fourth-degree polynomial. For grasslands, croplands and mature forests, we used a simple two-parameter mass balance model for SOC dynamics to fit the annual SOC stocks obtained by the application of RothC for each land class in each UHTU. The SOC balance is the difference between C input and mineralization, described by

$$\frac{dSOC}{dt}=K-\alpha SOC$$ (1)

where SOC is the SOC stock (t C/ha) at time t, K is the C input to soil at time t, and α is the C mineralization rate. C input (parameter K) is a function of the LU. Therefore, K and α are time-invariant and vary with location (i.e. UHTU) and LU class. Integrating between 0 and t, we obtain

$${SOC}_{t}=\frac{K}{\alpha }(1-e^{-\alpha t})+{e^{-\alpha t}SOC}_{0}$$ (2)

SOC is limited by an upper bound, i.e. SOC reaches a maximum attainable equilibrium, which is given by K/α. The curve fitting procedure thus provides attainable SOC stocks and mineralization rates that are LU and site-specific.

### Comparison of results and assumptions with data and previous uses of the model

Attainable SOC stocks calculated in this paper are difficult to validate for two main reasons. First, because they are regional averages valid at UHTU scale, which are relative large and approximately homogeneous areas. Our results should therefore be an approximation of measurements made at point/plot scale within UHTUs, but not an exact match. Second, they are difficult to compare with benchmarks because they are potentials rather than actual estimated SOC stocks. Comparison, for example, with available global SOC maps would be incorrect as those depict current SOC levels rather than potentials.

To assess the agreement between our results and field-level measurements of SOC, we therefore used a mixed strategy of indirect verification. Namely, we triangulated multiple lines of evidence to inquire whether literature or database sources lend support to our main results. This analysis included three separate parts.

First, we compared SOC stocks and mineralization rates obtained here with multiple field measurements taken from multiple croplands and forests in different locations of the world. In the absence of crop-specific global meta-analyses of SOC stock variation with LUC, we compare our results with prior local/regional applications of RothC. We compare results from those in-situ experiments to our results for the same regions, to assess if our approach, which is global and not tailored for farm-level assessments, can nevertheless adjust well to observations. However, to avoid comparing potentials (our work) with current SOC levels, we used only results from long-term experiments where, in principle, SOC stocks were closer to maximum attainable.

In this first step we used the search engine Google Scholar and the keywords “long-term experiment” + “soil organic carbon” and “long-term experiment” + “mineralization”. The search was conducted on July 30, 2018. We obtained 4,480 and 610 matches, respectively. We then selected only peer-reviewed studies published in books and journals with impact factor according to the 2017 InCites “Journal Citation Reports” from Clarivate Analytics [64]. We excluded project reports, Master and PhD dissertations and other grey literature sources. We also excluded experiments involving crop rotations or other LU changes within the study period, as these are not included in our analysis. As the goal was not to perform a comprehensive meta-analysis, we considered only the first 10 Google Scholar pages, i.e. 200 papers. We only used studies that explicitly report SOC stock (t C/ha) or SOC concentration (g C/kg soil) and bulk density (g/cm^3^) (to enable conversion of SOC concentration into stock). Using these criteria, we obtained a final list of 12 papers (out of 200).

The second comparison we made in this paper involved the model parameters chosen in local/regional and global applications of RothC regarding the DPM/RPM ratio and the decomposition rate of each pool. RothC was calibrated using SOC data from long-term experiments by the original developers of the model [30]. We used the default parameterization of the model for the global application. As parameters could be UHTU-specific, we checked whether other applications of the model have used non-default sets of parameters and, if so, if they were significantly different from the default. We again used the search engine Google Scholar and the keywords "Rothamsted Carbon Model" + "DPM" OR "RPM" OR "Hum" OR "Bio" OR "IOM". The search was conducted on December 2, 2018. We obtained 521 matches. We selected only peer-reviewed studies. Papers that used older versions of RothC than version 26.3 [30] and papers that only mentioned RothC without applying it were also excluded. Here, studies involving crop rotations were included. We included local/regional applications (mainly with measured soil C inputs) of RothC and continental/global applications (with estimated and aggregated data for soil C inputs). As the goal was not to perform a comprehensive meta-analysis, we considered only the first 50 papers (out of 521). Those 50 papers include a total of 233 applications of the RothC to particular points/regions. The full list of references, as well as parameters used in each of them, is included in S2 File.

The final step of this section of the work was a comparison of attainable SOC stocks obtained in this study with SOC concentration measurements from the LUCAS Project [17]. In this project SOC concentrations were measured at the 0–20 cm topsoil layer. To compare results, we assumed a uniform distribution on SOC in the topsoil profile, which means that 2/3 of SOC is in the 0–20 cm layer. Finally, to convert SOC concentrations (kg C/kg soil) to SOC stock (t C/ha), we used bulk density maps produced in the same project [65]. These site measurements covered 1,171 European UHTUs. As LUCAS assessed current SOC stocks, we could not compare values directly. Instead, we assume that higher present SOC should be, on average, correlated with higher potential SOC within the same land classes. We performed geospatial correlation analysis using Pearson’s r using 21 LU classes whose classification matches the system used in LUCAS. We excluded 20 LU classes because they had less than 100 observations each. To calculate these correlations, we used software SPSS version 23. We created a correspondence between the classification used in this study and the LUCAS Project classification, which is described in S1 File.

## Results and discussion

### Dynamics of SOC loss and accumulation

**Fig 2:**
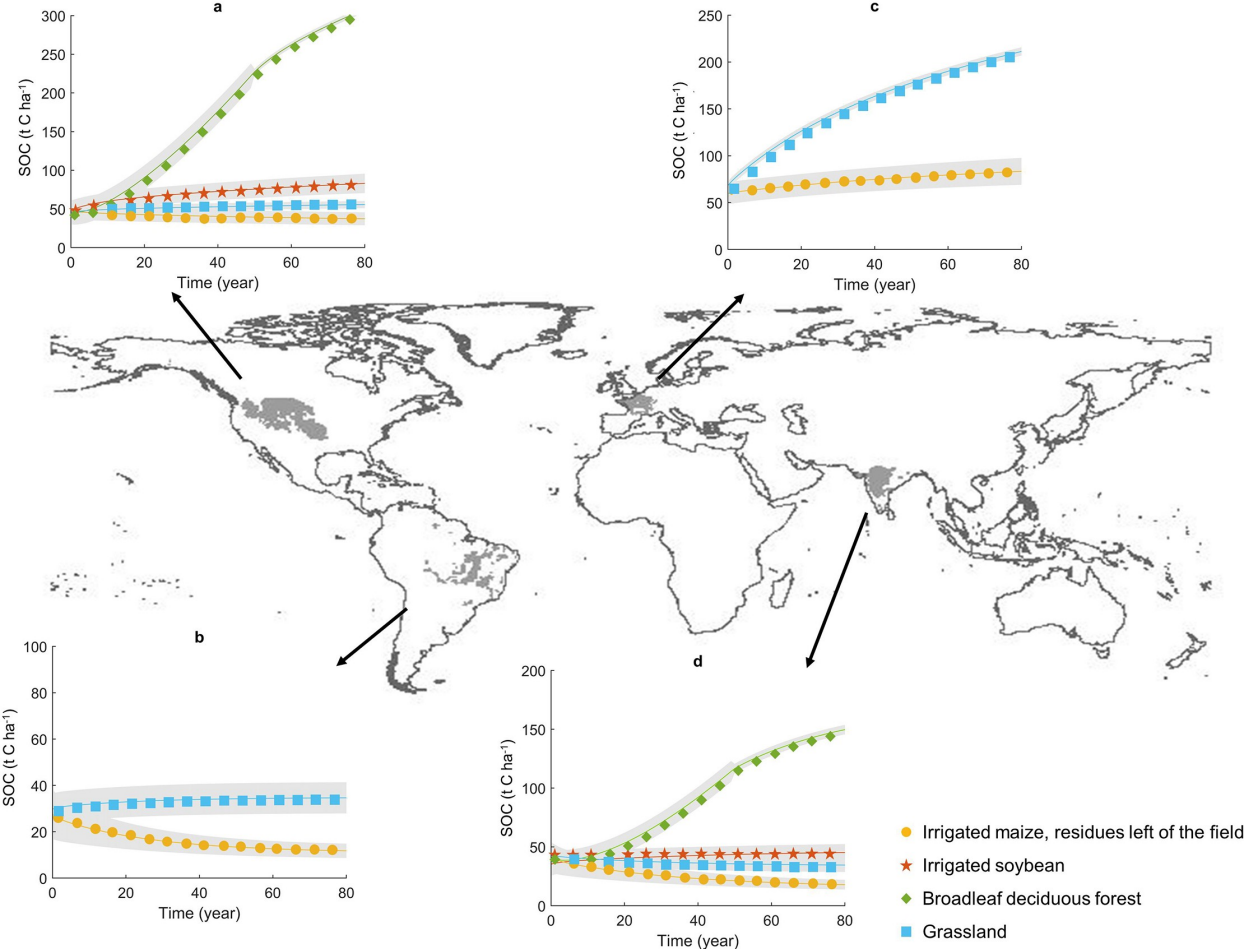
Soil organic carbon (SOC) stock (t C/ha) in four land use classes, in the scenario without manure application, in four unique territorial units studied (dark grey), namely: a, 911 (North America). b, 11867 (South America). c, 3499 (Europe). d, 7916 (Asia). Marks in the graphs represent mean annual SOC stock obtained in RothC, lines represent fitted exponential curves and the shaded area represents the 95% confidence interval of each fitted year.

We ran RothC for 86 years (from 2014 to 2100) and simulated SOC dynamics in all LU classes that are biophysically feasible in each of the c. 17,000 UHTUs, starting from present-day measured SOC stocks. Fig 2 displays selected results for the four largest UHTUs. SOC stocks (0–30 cm depth) are highly LU class-dependent even for classes that are traditionally aggregated such as different types of arable land. For example, in the region along the northern border of the United States of America (USA) with Canada the SOC stock after 86 years of irrigated maize with residues left on the field is 89 ± 3 t C/ha while for irrigated soybean it is 56 ± 2 t C/ha (Fig 2A), despite the fact that both are examples of typical agricultural classes. For each LU class, the SOC stock is highly dependent on the region under consideration: for example the average SOC stock for maize in Northwest India (Fig 2D) after 86 years is about 10 ± 1 t C/ha, which is considerably lower than the previously cited stock in North America (Fig 2A).

Forest LU classes such as “broadleaf deciduous forests” in North America (Fig 2A) and Asia (Fig 2C) need more time to reach the maximum attainable level. Immediately after transition to forest, SOC stocks decrease due to the low input of C residues as the forest is starting to grow. When forests reach a certain level of biomass stock, SOC begins to increase (Fig 2A and Fig 2D) as observed in other studies [5]. Climatic features such as temperature and precipitation and different soil types drive differences in soil C mineralization and also feasible yields, which in turn drives plant C inputs to soil.

**Fig 3:**
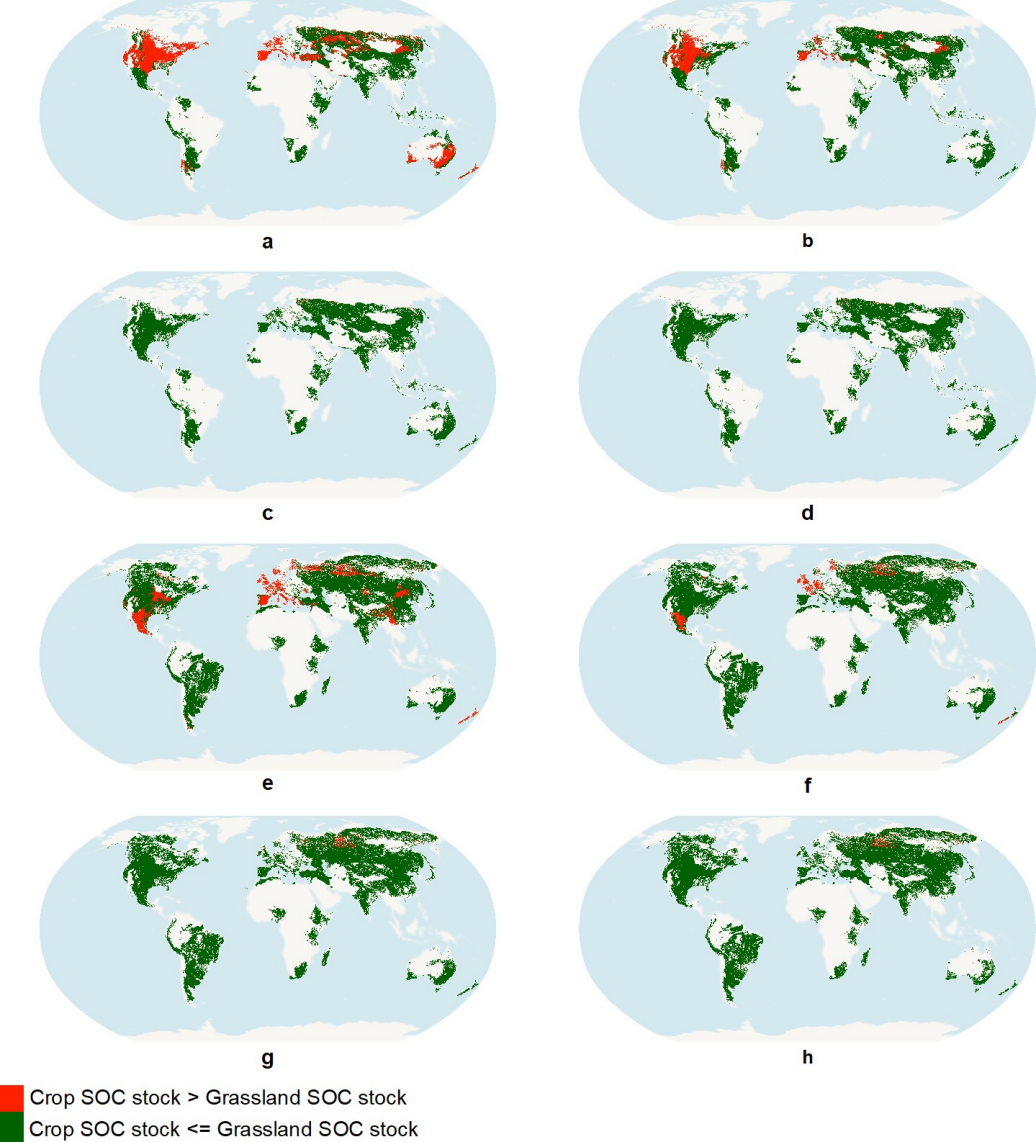
Visual representation of the regions where soil organic carbon (SOC) stock (t C/ha) is higher for grasslands than agricultural land uses (in green) or lower (in red). Agricultural land uses and practices represented: a, b, c, d–maize, e, f, g, h–wheat; a, c, e, g–irrigated, b, d, f, h–rainfed; a, b, e, f—residues left on the field, c, d, g, h—residues removed from the field.

In general, agricultural LU classes reach lower SOC levels than forests at maturity. Some boreal forests in western Scandinavia are exceptions where croplands can have higher SOC stocks than forests. Potential C input from residues kept on the field is high in these regions (e.g. tomato and maize), while boreal forests have low tree biomass and productivity and thus low soil C input [66]. Additionally, conversions to agricultural classes typically reach new SOC equilibria quicker than transitions to forest and grassland LU classes. Consequently, SOC loss is typically faster than SOC recovery. Grasslands generally reach higher SOC levels than croplands (Fig 2B and Fig 2D) due to plant shoot and roots and animal C inputs to soils [67,68], with notable exceptions in some regions for agricultural classes with high plant residues, such as irrigated maize (Fig 2A and Fig 2C). Fig 3 shows this effect clearly. When residues are removed from the field, croplands almost always reach lower SOC stocks, but if residues remain on the field there are large areas of North America, Western Europe, Central Asia and Australia where croplands accumulate more SOC than grasslands. Two effects justify this result–residues increase C input to soil, and soil cover with those residues reduces C mineralization. Out of the two, plant residue C input is the key explanatory variable of the results, but it is also the main source of uncertainty.

**Fig 4:**
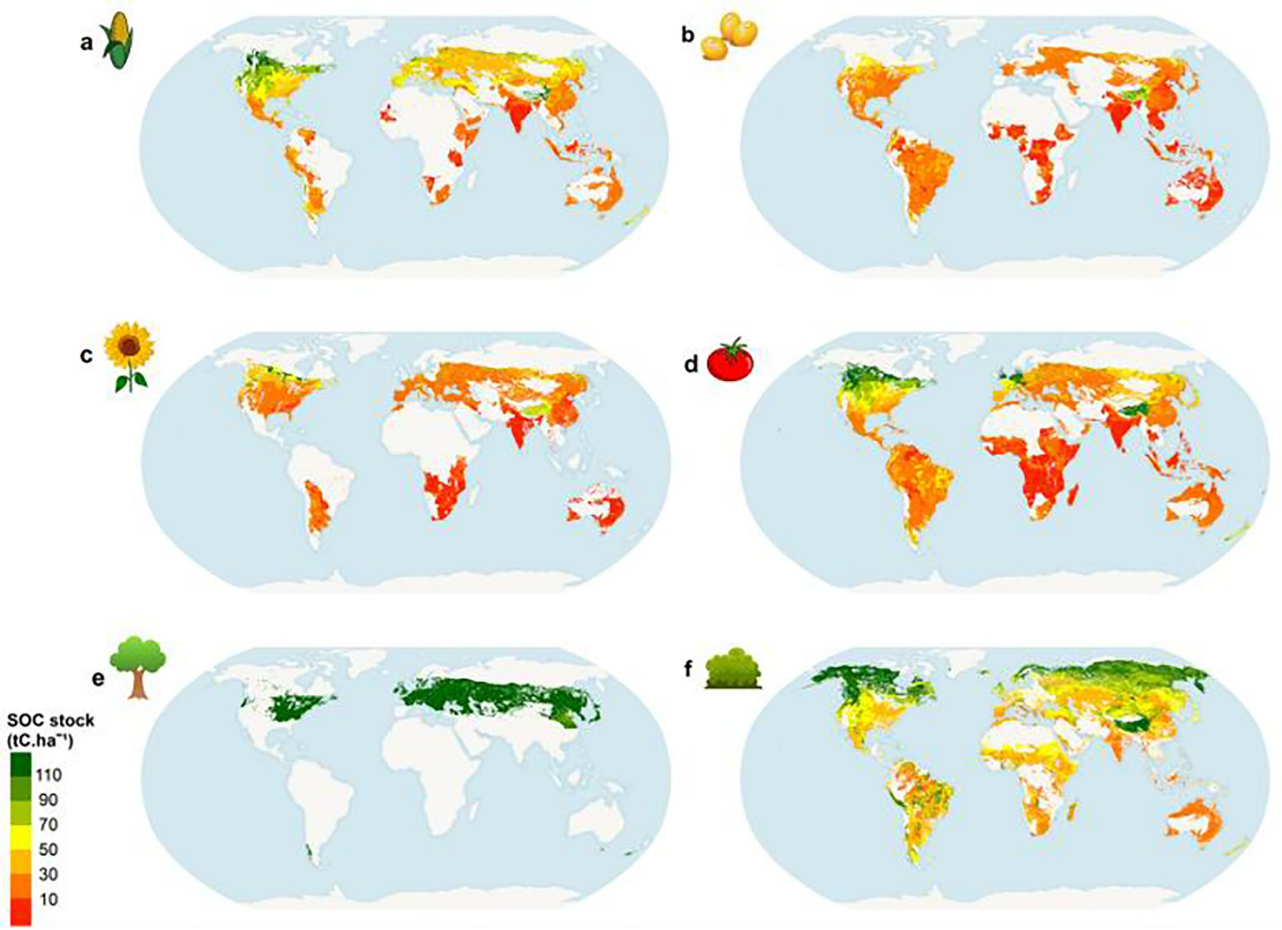
Attainable soil organic carbon stock (t C/ha) obtained by curve fitting per unique homogeneous territorial unit, in the scenario without manure application. a, irrigated maize, residues left on the field. b, irrigated soybean. c, irrigated sunflower. d, irrigated tomato. e, broadleaf deciduous forest in dry warm temperate zone, and. f, grassland.

In order to test the significance of results in Fig 4, we performed ANOVA for all UHTU and LU classes where attainable SOC for croplands were higher than attainable SOC in grasslands. On average, and for each LU class, the difference of the means is statistically significant at 5% in 30% of the UHTUs (15–67% depending on the agricultural LU class). Irrigated wheat with residues left on the field was the LU class with the highest number of UHTUs with p-value lower than 5% (165 out of 673 UHTUs), while rainfed sorghum with residues left on the field was the LUC class with the lowest number (2 out of 3). Irrigated maize with residues left on the field was the LU with the highest number of UHTU where attainable SOC was higher (625 UHTU), but the difference is only statistically significant in 20% of those UHTUs. These tests reassure statistically that for a fraction of the cases identified in Fig 4 some croplands indeed attain higher SOC.

### Global distribution of long-term SOC equilibria and mineralization rates

To aid in interpretation, we fitted an exponential model to each 86-year SOC time series, depicting inter-year changes in SOC stocks as a simple balance between fixed C inputs to soil and fixed rate C mineralization and emission as CO_2_ through soil respiration; this is effectively the most simplified version of RothC possible, considering a single C pool in the soil and a mean rate of mineralization for the average pool. The upper limit of the exponential model is the maximum SOC stock obtainable, or attainable SOC. The full list of Curve fitting results (i.e. long-term SOC stock, mineralization rates and the parameter K) are presented in S4 File.

**Fig 5:**
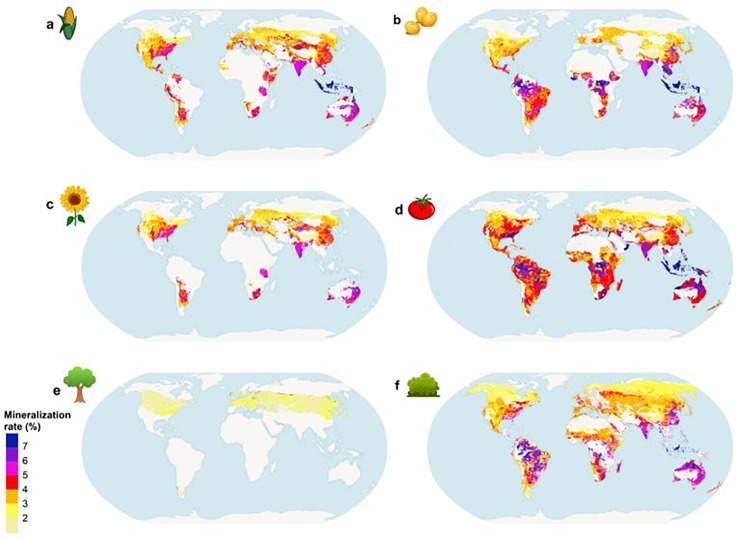
Average organic carbon mineralization rate (% per year) obtained by curve fitting per unique homogeneous territorial unit, in the scenario without manure application. a, irrigated maize, residues left on the field. b, irrigated soybean. c, irrigated coconut. d, irrigated tomato. e broadleaf deciduous forest in dry warm temperate zone, and. f, grassland.

Fig 4 depicts the global distribution of attainable SOC stocks for six particular LU classes, while Fig 5 shows C mineralization for the same classes. Note that none of these classes exist in all UHTUs, because of biophysical feasibility constraints. Irrigated tomato production, for example, is feasible in 11,985 UHTUs (the highest number for all LU classes), and rainfed oil palm in 73 UHTUs (the lowest number).

Regardless of LU class, high latitude regions and (for some land classes) Equatorial regions display higher attainable SOC stocks (Fig 4), but for different reasons. Equatorial regions are marked by high temperature and high rainfall, causing higher soil respiration (Fig 5) through the effect on soil microbial activity [69,70], but this effect is countered by higher yields and consequently higher plant C input to soil. High latitude regions have high water availability and low temperature, and therefore low mineralization rates (Fig 5). The Mediterranean and Australian semi-arid regions show high mineralization rates, but attainable SOC stocks are higher along the Mediterranean due to higher plant C inputs to soils (e.g. Fig 4D and Fig 5D).

Coniferous forests in northern boreal regions (Fig 4E) have the highest attainable SOC out of all types of forest anywhere in the World. Partially this is the result of climatic conditions in the north boreal regions, but also of high plant C inputs. Southern boreal forests have lower attainable SOC than northern regions as mineralization rates increase towards the Equator. Despite lower mineralization in high latitude regions (boreal thermal zones), these forests in the Southern border tend to have lower attainable SOC stock compared with temperate forests due to higher annual litter production of the latter. In UHTUs where boreal coniferous forest and temperate continental forest can coexist, the latter have on average 75 t C/ha higher attainable SOC.

Regarding grasslands, the input of C from dung deposition depends on grazing intensity, which introduces a high degree of spatial variability in attainable SOC. Lower SOC stocks are found in arid and dry areas of Oceania (particularly Australia). In general all LU classes in this region have low attainable SOC stocks (Fig 4) and high mineralization rates (Fig 5). Grasslands have particularly high SOC stocks in equatorial regions (Sub-Saharan regions, Central America and Southern Asia regions), which is 7 t C/ha higher than in croplands on average. However, croplands with high residue production crops (e.g. maize, wheat) in mid and high latitude regions, can have stocks up to 20 t C/ha higher than grasslands.

For maize (Fig 4A) there is a downward North-South gradient. In North Canada and the USA SOC stocks are higher than in southern USA and Mexico, although moving towards the South of the continent SOC increases slightly for some crops and grasslands. Typically, attainable SOC stock in Africa is lower than in the other regions due to high turnover rates of organic material combined with low yields [71]. Irrigated sugarcane is the technically feasible crop most found in African UHTUs, but it is also the agricultural LU class with the highest mean mineralization rate (about 5% per year). Tropical and subtropical forest classes have lower mean SOC mineralization rates (about 1% per year) than arable land classes.

**Fig 6:**
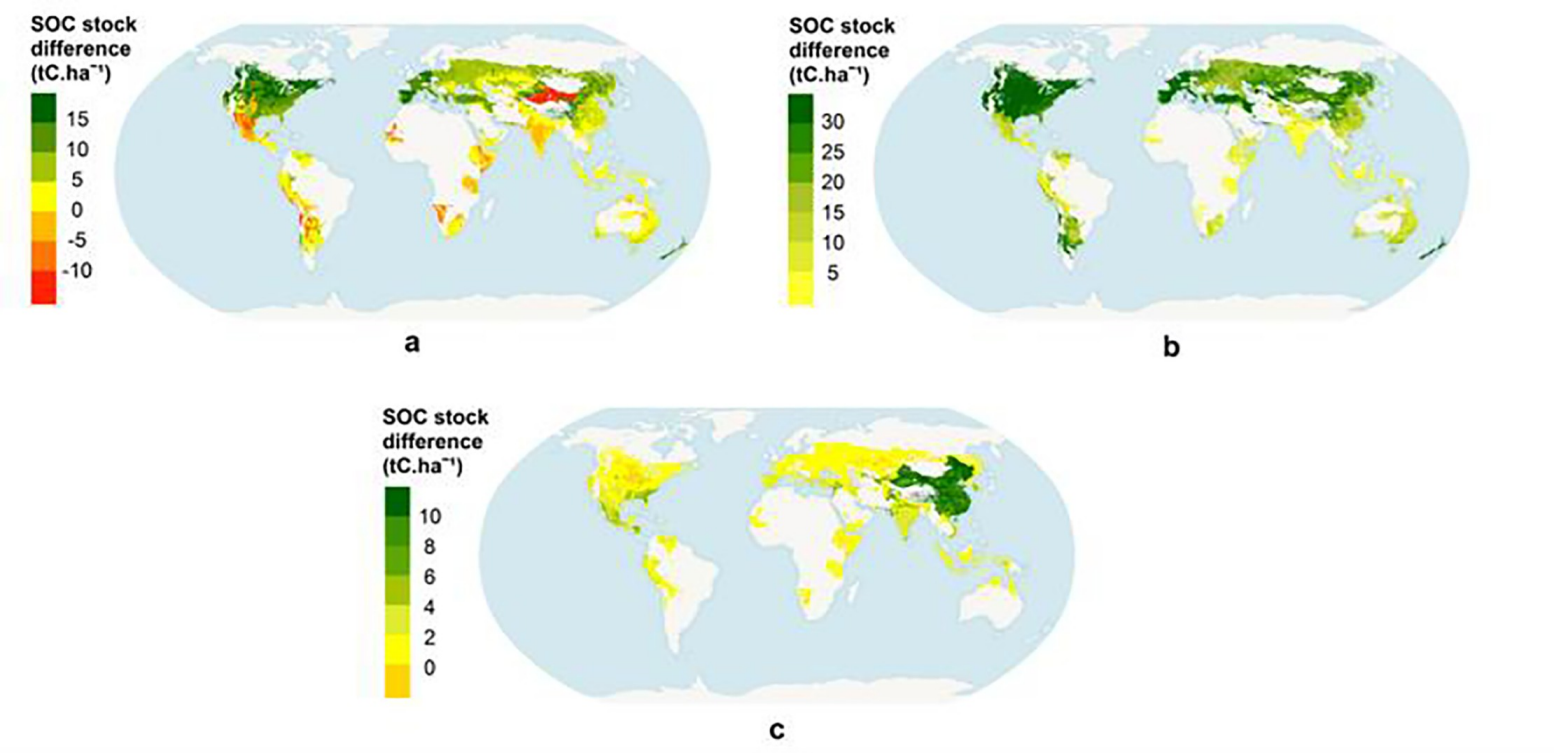
Soil organic carbon (SOC) stock difference (t C/ha) for the effect of management practices on attainable soil organic carbon stock for maize. a, SOC for irrigated maize minus SOC for rainfed maize. b, difference in SOC for maize with residues left on the field and with full extraction of residues. c, difference in SOC for 100% organic fertilization of maize and 0% organic fertilization.

Fig 6 depicts the impact on attainable SOC stock of management practices in grain maize production. We tested: a) the effects of irrigation against rainfed maize, b) leaving plant residues on the field against exporting crop leftovers, and c) organic fertilization. The distribution is regionally asymmetrical, but in general leaving residues on the field (Fig 6B) produces higher increases of attainable SOC stock than irrigation (Fig 6A) and organic fertilization (Fig 6C). The average effect of irrigation on all agricultural classes is an increase in attainable SOC of 4 ± 2 t C/ha. Irrigation has less effect on permanent crops (0.9 ± 0.1 t C/ha). Wheat is the agricultural class where irrigation has a higher positive effect (10 ± 5 t C/ha). SOC increased the most with irrigation in temperate thermal zones. For example, the SOC increase due to irrigation at temperate oceanic regions is 3–8 t C/ha for UHTUs. On average organic fertilization increases the attainable SOC stock of agricultural classes by 3 ± 1 t C/ha. Potato (irrigated and rainfed) is the class where organic fertilizer application increases attainable SOC stock the most (about 8 ± 3 t C/ha). Temperate thermal zones are where organic fertilizer application increases SOC stocks the most, between 4 and 6 t C/ha. Maintenance of residues on the field increases attainable SOC stock for cereals classes by 12 t C/ha. The increase is highest (18 ± 4 t C/ha) for maize and wheat.

Sugar crops (sugarcane and sugar beet) and tomato have the highest attainable SOC stocks among crops, due to higher yields and production of residues. These crops are estimated to produce residues that are twice the yield. Others crops, like tobacco, also produce more-than-proportional residues but have low yields and as such do not accumulate as much SOC. Irrigated sugar beet leads to, on a global average, 20 t C/ha higher stabilisation SOC compared with irrigated maize with residues left on the field. In this case, although maize is more productive, the fraction that is plant residue is much lower.

Additional management practices such as tillage can potentially affect SOC stocks, but are not explicitly modelled by RothC. In the literature, the effect on SOC stocks of replacing conventional tillage with no-till is on average 2–10 t C/ha [72,73]. According to the IPCC [19], tilled cropland soils have 2–15% less SOC than no-till soils. This is less than half (31%) of the difference between attainable SOC stocks of grasslands and cereals found here. Our conclusions about regions where croplands reach higher SOC stocks than other land uses are therefore not affected by omitting the effects of tillage.

### Comparison of results with data

Global modelling exercises provide highly uncertain results, in particular for SOC, which is highly sensitive to geography and dependent of LU class. Our application of RothC provides results depicting trends at UHTU level. At higher scales, results are well aligned with prior literature, despite high variation due to the crop-specificity of our results. For example, in Fig 2C the SOC gain due to transformation to forests depicted for temperate zones is about 200%. Prior research estimated SOC stock gains between 75% and 200% for transitions from cropland to forest (after 100 years), also for temperate zones [5]. Nevertheless, despite the high level of regionalization, these estimates are not necessarily farm-specific and are not applicable locally. This raises the question of whether the outputs of the model realistically depict SOC as measured.

In general, attainable SOC stocks and mineralization rates reported here are similar to values reported in long-term field measurements for specific sites reported in other studies. In the Huang-Huai-Hai Plain of China [74] the long term SOC stock (without organic fertilizer application) at 20 cm depth for maize production is 18 t C/ha (27 t C/ha, assuming homogenous distribution in the first 30 cm depth). Our results for the same region were 20 ± 2 t C/ha (30 cm). In another Chinese region (Jiangxi) [75] long-term maize production without fertilization results in a SOC stock of 20 t C/ha, which changes to between 23 and 31 t C/ha with different fertilization rates. For the same region we obtained 25 ± 5 t C/ha and 38 ± 8 t C/ha, respectively. In a Canadian region [76] SOC in wheat fields stabilizes at 35 t C/ha (at 15 cm depth– 70 t C/ha at 30 cm) when straw is left on the field, while we report 51 ± 10 t C/ha for the same case and region. In the Harpenden region (United Kingdom) [30] SOC stocks of about 25 (at 15 cm depth– 50 t C/ha at 30 cm) and 60 t C/ha (at 23 cm depth– 78 t C/ha at 30 cm) were reported for arable land and mixed deciduous forest following a calibration procedure of RothC using field-level data. We obtained for the same region 35 ± 9 and 66 ± 15 t C/ha. In the Ruzyně region (Czech Republic) [30] arable land has 32 t C/ha in the first 20 cm (48 t C/ha at 30 cm), according also to a calibration study with the same model, while we obtained 29 ± 11 t C/ha. In the Halle region (Germany) [77] maize production without fertilizer applications leads to the accumulation of 59.7 t C/ha and 43.2 t C/ha (at 35 cm depth– 51 and 37 t C/ha at 30 cm), while in the same region we obtained 49.6 t C/ha and 48.6 t C/ha at 30 cm, respectively.

This assessment shows that, discounting differences in sampling depth, our results are generally in agreement with measurements. In particular, there is good agreement for European temperate regions, as the default parameterization of RothC used here was obtained for those conditions (originally for measurements of SOC in Harpenden, United Kingdom [30]). However, this is not the case for all regions of the World. Despite the notable exceptions mentioned earlier, the estimation agreement is lower in regions with significant different climate conditions to those where RothC were calibrated. Estimated attainable SOC for wheat in three Indian regions (Barrackpore, Ranchi and Akola) [78] were 40% lower on average. SOC stocks for wheat in those regions (at 30 cm depth) of 39, 24 and 19 t C/ha were reported. Respectively for the same three regions we obtained 17 ± 4, 15 ± 5 and 14 ± 2 t C/ha. In the Sidney region (Australia), long-term grasslands reached between 58 t C/ha and 82 t C/ha (depending on the tillage practice). In our work, we obtained 38 ± 3 t C/ha. Long-term rice cultivation in South Korea leads to a SOC stock of 60t C/ha (without fertilization) and between 60 and 69 t C/ha, depending on the fertilization rate, while we obtained 30 ± 6 t C/ha and 32 t C/ha, respectively. Regarding the mineralization rate, an average range reported for sorghum production in Burkina Faso [79] is 1.5 to 2.6%, which is similar to 3 ± 1% obtained in our results. The differences can be justified due the differences in climatic variables between studies (e.g. the average rainfall used in this study is 200 mm higher). The average rate for fertilized grasslands in England [80] are 3.0%, compared with 3.7% in our study. For oak forests in Italy [81], mineralization rates reportedly range between 1 and 6%, while for the same region we obtained an estimated 5 ± 1% for temperate oceanic forests. As for the role of management practices on SOC, for maize production in a Loess Plateau (China) [82] leaving straw on the field increases annual SOC stock in 0.150 t C/ha.year. For the same region we obtained 0.350 ± 0.035 t C/ha.year, considering the difference in SOC between the first year and at the end of the 86-year simulation. A study in central Ohio (USA) [83] calculated that wheat residues left on the field increase SOC by about 3 t C/ha.year. For the same region, we obtained 2 ± 2 t C/ha.year on average for all LU classes.

A systematic comparison of our results with individual studies would require a large meta-analysis of SOC measurement studies, which is outside the scope of this study. Our results are valid over relatively aggregated regions that are approximately homogeneous (i.e. UHTUs), but the studies in the literature are valid for specific points within those regions. It would be necessary to aggregate the results of those studies into broader areas in order to compare them with our regionally applicable results. At the moment, these comparisons provide promising indications that our results depict the main trends when comparing regions and LU classes. This provides some qualitative assurance for the main insights obtained from the results in this paper. In most cases where the literature reports measured SOC stocks that are significantly different from our results, the relative comparisons between LU classes and locations are very rarely different. If a given LU class in a given region has a higher measured SOC stock than another LUC class in another region, our model results typically also report a higher SOC stock.

Besides individual studies, we also compared our results with pre-established SOC databases. This enables a systematic assessment of the agreement between results. We used the geo-referenced 19,000-measurement LUCAS database for the EU [17]. A geospatial analysis of LUCAS depicts the same general conclusions of our work. Considering only EU countries, the regions with higher attainable SOC stocks are Northern Europe, Southeast France and the British Isles, which are the regions where higher SOC field measurements are located in LUCAS. This fact can be explained by the combination of three factors: temperatures are relatively low temperate, precipitation is relatively moderate (in the case of British Isles) or low (Southeast France) and organic soils, which are typically high in SOM [17,84,85], are present. Results of geospatial correlation analysis shows that for 13 LU classes out of 21 evaluated SOC stocks in our work and LUCAS measurements are significantly correlated at 5% (mean Pearson’s r is 0.212). For sugar beet and rapeseed no spatial correlation was found. This result is due to the fact that our results depict attainable SOC stocks, while LUCAS measurements depict SOC stocks at an unknown time after transformation, with no assurance that SOC at each location has stabilized. The fact that a positive correlation was found is reassuring, as a stronger correlation could not be expected.

### Comparison of assumptions with previous uses of the model

Regarding the choice of parameters made in previously published works using RothC at point/plot scales, we observed a widespread use of default DPM/RPM ratios and decomposition rates for the different organic matter pools (Table 1). Most model applications (197) use only defaults (i.e. DPM/RPM ratio of 1.44 for cropland and improved grasslands, 0.67 for unimproved grassland and scrub and 0.25 for forest; and decomposition rate of 10, 0.30, 0.66, 0.02 1/year for DPM, RPM, HUM and BIO, respectively). The decomposition rate of the HUM pool was the parameter most often re-parameterized, followed by the DPM/RPM ratio of the soil C inputs. The HUM pool is most often re-parameterized (as for example [86–88]) as it has the most sensitive kinetic constant (due to the longer residence time). Regarding land classes (data shown in S2 File), all three main LU types (cropland, grassland and forest) are covered by the sampled papers, and for each of them multiple sub-classes are considered—e.g. in the cropland class, the studies assessed involve 20 different crops/rotations (among others, maize, wheat and rice). Forest is the class that required more re-parametrizations (23 out of 52 applications), mainly in the HUM decomposition rate. Nevertheless, twenty-two of those re-parametrizations are from only one study (Shirato et al. [86]). Croplands required the least changes (only 5), which was expected considering that RothC was originally developed for croplands. This low number of re-parameterizations in studies that applied the model at very fine scales provides some assurance that the default configuration of the model used in this work works well for most regions of the World.

**Table 1:** Accounting of the RothC model parametrization. BIO microbial biomass; DPM—easily decomposable plant material; HUM—humified organic matter; RPM—resistant plant material.

Was it changed?	DPM/RPM ratio	Decomposition rate			
		DPM	RPM	HUM	BIO
Yes	10 (4%)	2 (1%)	4 (2%)	36 (15%)	1 (<1%)
No	222 (96%)	231 (99%)	229 (98%)	197 (85%)	232 (>99%)

The resilience of our results is also demonstrated when comparing our results to results obtained using different parametrizations for specific regions. For example, RothC was originally developed for humid regions and is known to overestimate C mineralization in dry regions [89]. A small change in the soil moisture rate modifier was proposed (where the minimum value of the rate modifier was changed to 0.1 –originally it was 0.2) [89]. Comparing the attainable SOC stocks in a dry region (Alentejo, Portugal) that were obtained in a previous regional study [33] using the modified version of RothC to the attainable SOC stocks obtained in this paper for the same region (using the “default” RothC parameter set), there are no statistically significant differences in results (considering the confidence intervals for both applications of the model). For example, the average attainable SOC stocks for irrigated maize with residues left on the field obtained in this paper was about 38 ± 7 t C/ha, while in the regionally modified application it was 41 ± 4 t C/ha. The average obtained in each model application for each land class always falls within the confidence interval obtained in the other. Therefore, despite of the reported overestimation of C mineralization under dry conditions, there is no statistically significant difference in results for our application at UHTU scale.

### Comparison of results with large-scale RothC applications

We only found 2 continental/global applications [32,36] of RothC in the first 200 references (out of the 521). As in this paper, all of them used the model without re-parametrization. Those studies include the main three LU classes, cropland, grassland and forest, without discriminating between sub-classes (e.g. specific crops). For grasslands, the model application performed by Smith et al. [36] at European scale considered a DPM/RPM ratio of 1.44 (i.e, the default for improved grassland), while the global application performed by Gottschalk et al. [32] considered 0.67 (i.e. the default for unimproved grasslands). Our study is therefore aligned with past practices in terms of parameter selection but adds significant regional and LU detail when compared to those prior applications. Both papers focus on the effects of climate change and estimate a SOC loss in cropland and grassland areas in Europe due to warmer temperatures. They also note that SOC loss may be slowed by decreased soil moisture. Those results cannot be directly compared to the results obtained in this paper because our study was not forward-looking and did not take the effects of future climate change (temperature and precipitation) into account. However, our results have implications for studies aimed to estimating the effect of climate change on SOC loss. We show that SOC loss/gain is highly related with the specific crop type. In the same UHTU, land occupation with one crop type could lead to SOC gains and with another crop to SOC loss due to different yields and their response to changing climate conditions. These results therefore suggest that future similar studies should consider at minimum the same level of differentiation between crop types and regions used in this paper.

The use of a Monte Carlo approach in this work also had additional value as it avoided assuming that the number chosen for each input variable was representative of each entire region. Input data varied per iteration according to a pre-set probability distribution depicting the regional variability of the data within each UHTU. The average of the 100 iterations performed for each UHTU and land class, which is indicated in this work as the most likely result (with accompanying uncertainty intervals), is therefore representative of the UHTUs they depict.

### Limitations, extensions and future work

#### Comparison of RothC with other models

We selected RothC for this paper, but RothC is one of several soil process-based models available that could have been used instead. Among those, two notable models that were considered as alternatives for the research conducted here were the widely used CENTURY [90] and DNDC [91] models. Originally, all models simulate the first 20 cm depth, but all were already used at 30 cm depth. All models run using a monthly time step. CENTURY has a broader scope than RothC, including not only soil C, but also N, phosphorous and sulphur dynamics. DNDC includes the C and N cycle in the soil. Having a larger scope for modelled processes, a limiting factor for using CENTURY and DNDC in full is the higher number of variables/parameters required. Some variables are difficult to obtain for a global application with the characteristics of the one in this paper. We focused specifically on processes influencing C dynamics, and, as RothC includes only the C cycle, the number of parameters involved and data required is lower. The lower number of required input data makes a spatially differentiated and crop-specific global assessment feasible. Higher computational time requirement to run CENTURY and DNDC are additional limiting factors to use these models at global scale.

For C, RothC considers a total of five pools (two litter pools, DPM and RPM, and three soil pools, HUM, BIO and IOM), while DNDC has four pools (plant residue, microbial biomass, active humus and passive humus) and CENTURY includes three SOM pools (active, slow and passive) plus two litter pools (structural and metabolic) that are not included in the soil. The simplicity of RothC, however, comes at a cost as it does not model some processes. It is the only of the three models that omits leaching of organic matter and that does not include a management practices module (other models can simulate the effects of fertilization, harvesting, fire, grazing, irrigation and erosion–in some cases through extensions or more recent modules built on top of the original model). The lack of depictions of these processes are limitations that may be overcome through extensions to RothC and are suggested for further revisions of work similar to what we present in this paper.

#### Model parameterization

Despite several limitations inherent to the task of performing global SOC modelling, such as the simplified depiction of soil biogeochemical processes by the model and uncertainty of the input data (e.g. plant residues), our results qualitatively depict well-established regional and global trends, such as the fact that SOC stocks are lower for agricultural LU classes and that SOC loss in transitions to croplands is a faster process than SOC recovery. Our results are also robust to differences within each LU class, e.g. attainable SOC stock for wheat production is higher in northern Europe than southern Asia [76,78]. Here, we used a “generic” parametrization of the RothC model, which together with other factors determines the obtained attainable SOC stock and mineralization rates. It is likely that general estimation accuracy could be improved using local parametrization of the model, as performed by some prior RothC applications [86–88], for those regions and classes where the estimated results deviate from field-measured values. We expect locally specific parameterizations to improve results because the agreement of the model with other published studies seems to be better for temperate regions closer to the ones used for the original calibration of the model. The quantitative comparison of results with data presented here is also promising despite the fact that more work is needed to assess the regions and classes for which the model works properly and the ones where the model fails to depict realistic SOC stock dynamics.

Attainable SOC stock and mineralization rates are also influenced by other methodological choices done in this work. The Monte Carlo determination of intra-UHTU uncertainty did not take into consideration the uncertainty in RothC parameters. Only input variables had an assigned probability distribution within each UHTU, but even for those we assumed as a simplification that all input variables were normally distributed. When we obtained the data from a geospatial dataset (i.e. we estimated the parameters for a normal distribution starting from pixel-level data within each UHTU), we verified statistically that the assumption was plausible. For some data, however, we were unable to do so as we had to assume a distribution based on one likely value plus a confidence interval (e.g. IPCC-based data on yields). It is unlikely that all input variables are in fact distributed normally. For example, clay content is frequently used in modelling according to a normal distribution (e.g. [33]), but empirically that is rarely the case [92]. This information influences the depiction of intra-regional variability through the uncertainty quantified using Monte Carlo analysis.

#### Calculation of soil carbon inputs

Soil C input is a critical factor in explaining differences between attainable SOC, as demonstrated by complementary analysis reported in S1 File. Yield data (FAOSTAT [44]) and the factors used to calculate above and belowground residues (IPCC [19,42]) contribute the most to the soil C inputs and their uncertainty. In this work, for all agricultural LU classes, we considered constant plant yields, as well as constant aboveground and belowground residue C inputs and C from manure application.

This method introduces error in cases where belowground plant growth does not scale with aboveground yield. There is some evidence that belowground C varies less between crops than aboveground C. For example, Cagnarini et al. [35] conclude that the use of constant factors overestimates belowground biomass when compared with field-measured belowground biomass, for maize and wheat in Switzerland. Taghizadeh-Toosi et al. [93] used long-term experimental data to conclude that belowground biomass growth was independent of the crop. In general, the literature overall shows that the type of crop influences belowground biomass (e.g. [42,94,95]), but belowground C is also highly influenced by the year and farming system (e.g. conventional or organic) [95]. The method used in this paper prevented us from simulating intra-annual changes in crop residue C inputs from belowground as we use constant near-past average monthly temperature and precipitation. Studies that, unlike this one, are interested in understanding the dynamics of SOC gains and losses due to climate change or other dynamic processes should take into account that belowground biomass can be nearly uncorrelated with the response of the aboveground biomass, which is typically easier to estimate.

Additionally, in order to simplify the calculation of C inputs, we also considered the same C content of the crop, forest and grassland residues (i.e. 0.40 kg C/kg DM) and constant C/N ratio of manure applied (i.e. 14 kg C/kg N). While there is good evidence for the C content of living plant biomass [19], there are many types of manure with varying chemical compositions [96]. Due to lack of data, we were unable to consider specific types of manure applied in each UHTU, or their quantities. As future work, a sensitivity analysis should be performed for those critical parameters.

#### Depiction of agricultural management practices

A remaining significant limitation is that the model was unable to assess a wider variety of management practices. For example, this work does not yet include an assessment of SOC changes in no-till systems or crop rotations, as well as the influence of forest fires [97]. For woody crops, an evaluation of the differences between bare soil and use of cover crops is also missing. All these effects are crucial and deserve inclusion in future models or iterations of this work. Another limitation of this work is that only one grassland class was included, despite the multitude of grazing systems and respective management practices in the World. Follow-up work should involve multiple grassland classes, as well as the explicit modelling of one or more shrubland classes. Having multiple grassland types (and also grassland management) and expanding the scope of cropland management practices should also enable more comprehensive comparisons of attainable SOC stocks within and between LU classes. Within classes, it will produce results regarding the most SOC-increasing practices in each UHTU. Between classes, it will produce updates results regarding where in the world and for which combinations of practices particular cropland can accumulate more SOC than grassland or forest.

At the moment the IPCC [19] only provides SOC stock estimates (under native vegetation) for a combination of 9 climate regions and 6 soil types. To calculate SOC stock for other classes (e.g. long term cultivated land), the IPCC method applies simple management factors to the estimated SOC stock under native vegetation depending on climate region, moisture regime and tillage type. However, this procedure is not crop-specific and limited in biogeographical differentiation. We thus consider that the use of the results provided in this study is a significant step forward that can provide an alternative, more accurate quick assessment tool. These results can be used for large-scale assessments of SOC change in the absence of local information, such as Tier 2 IPCC defaults [19] involved in the calculation of C sequestration and C emissions due to LUC.

#### Potential extensions of the work

The data in this paper also enables the historical assessment of SOC gains and losses due to past land use change, as well as prospective studies for scenario assessment of future land conversions. For example, studies that assess the effects of land use in the recent past on SOC can use the data presented here to estimate the sign and magnitude of the difference before and after changes. Average mineralization rates per LU class and region can also be used for additional modelling exercises.

A different application of the work presented here is in Life Cycle Assessment (LCA) studies. There has been a push in recent years to produce accurate and meaningful LCA impact assessment models determining the impacts of land use [98–102]. For the role of land use on the loss of the soil’s biotic production potential, SOC depletion is commonly used as a proxy indicator. After the original models based on IPCC data [103,104], more advanced models using statistical interpretation of global SOC maps have been proposed [84,105]. These models are very limited by data availability and end up depicting a very limited number of land classes or miss critical biogeographical differences [105]. Process-based modelling of SOC can overcome those limitations and provide accurate and highly detailed LCA characterization factors. A demonstration of the potential of using RothC for this end was published for the region of Alentejo, Portugal [33]. The data presented in this paper enables the extension of that work for the World.

## Conclusion

This work provides a new global SOC assessment enabled by process-based modelling rather than conventional statistical upscaling of local measurements. Global modelling is not new, but here we introduce important innovations in SOC modelling regarding the depth (number of land classes) and reach (spatial representation) of the analysis. We calculated annual and attainable SOC stocks for 80 combinations of LU and management practices in approximately 17,000 regions in the World. This study therefore significantly increases simultaneously the number of LU classes and the spatial resolution of modelling, compared to other global estimations of potential global SOC stocks.

We conclude that using aggregated “cropland”, “agriculture”, “arable land” or other similar land use or land cover classes in global assessment is too coarse to produce meaningful results, as demonstrated by the extreme variability of results for each cropland sub-class within the same region and between regions. We showed that this approach to modelling can produced novel results absent from past modelling exercises. For example, we also showed that, by performing this differentiated, crop-specific analysis, some crops in particular regions of the World can accumulate more SOC than forests and grasslands. This effect exists mostly when crop residues are left on the field, which has a double effect: the amount of C entering the soil is higher, and the soil remains covered for a longer period of time, reducing aeration and mineralization. These are particular cases that do not invalidate the generic observation that conversions to cropland overall reduce SOC, but they should be acknowledged as those exceptions are relatively frequent and exist even for highly important and representative crops such as maize.

## Supporting information

S1 File Supplementary methods, results and future applications. (DOCX)

S2 File Comparison between model results and long-term experiment data. (XLSX)

S3 File Unique Homogeneous Territorial Unit (UHTU) map. (DOCX)

S4 File Curve fitting results. (DOCX)
