# Supplementary material for: Detailed global modelling of soil organic carbon in cropland, grassland and forest soils
Source: PLoS One. 2019 Sep 19;14(9):e0222604. doi: 10.1371/journal.pone.0222604 (PMC6752864; doi:10.1371/journal.pone.0222604)
Supplement: S1 File — (DOCX) [file pone.0222604.s001.docx]

Detailed global modelling of soil organic carbon in cropland, grassland and forest soils

Tiago G. Morais, Ricardo F.M. Teixeira and Tiago Domingos

**Supporting Information File S1**

1. **Supplementary methods**
   1. **Additions to the standard RothC model**
      1. Dung during grazing

In this work, we used The Rothamsted Carbon Model (RothC) [1]. Fig. 1 shown the schematic distribution of soil organic carbon (SOC) through the compartments used in RothC. The five soil organic pools and respective interactions are represented, easily decomposable plant material (DPM), resistant plant material (RPM), microbial biomass (BIO) and humified organic matter (HUM) and inert organic matter (IOM). The IOM pool is resistant to decomposition and does not receive C inputs. This model was originally developed for application to croplands and it has also been successfully applied to forests (Hashimoto and collegues [2]) and grasslands. In some RothC applications to grasslands dung during grazing from the animals is omitted [3,4]. We modified the model to also include animal-based carbon inputs. We assumed that the partition between SOC pools for dung during grazing was similar to the partition for farmyard manure, i.e. 49% were assigned to easily DPM pool and RPM pool, and the remaining 2% went into the HUM pool.


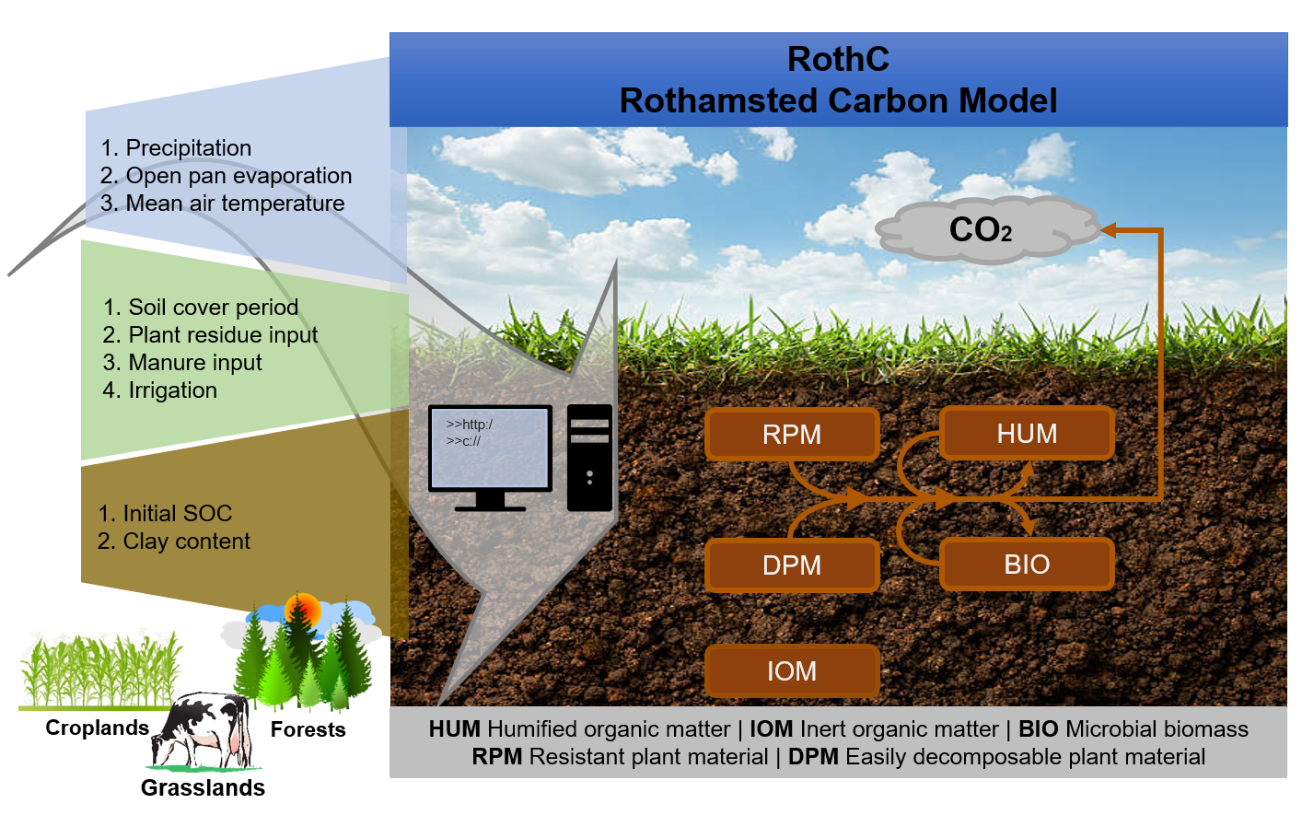


**Fig. 1.** Schematic representation of the RothC model, depicting the inputs required and structure of pools - adapted from the RothC manual [37]**.**

- - 1. Uncertainty

To obtain uncertainty estimates for the outputs of RothC, we used a Monte Carlo approach [5], where for each Unique Homogeneous Territorial Unit (UHTU) the model was run one hundred times. Each iteration used a unique set of inputs drawn randomly from the respective normal probability distribution. Table 1 summarizes uncertainty sources for all model inputs. In the next section, we will detail the method used to obtain other inputs and respective probability distribution except for initial soil organic carbon (SOC) stock and clay content, whose only uncertainty source was spatial variability in each UHTU.

**Table 1.** Uncertainty sources summary. Unique Homogeneous Territorial Unit (UHTU).

| **Model inputs** | | **Uncertainty Source** |
| --- | --- | --- |
| Initial condition | Initial soil organic carbon | Data source (map)’s spatial variability per UHTU |
| Soil parameter | Clay content |  |
| Plant residues | Croplands | Yield time series (2004-2014) |
|  |  | Parameters obtained according to the method in section 1.2.1.1 |
|  | Forests | Parameters obtained according to the method in section 1.2.1.1 |
|  | Grasslands | Obtained from IPCC (2006) [6] |
| Farmyard manure | | Spatial variability per UHTU |
| Dung during grazing | | Spatial variability per UHTU of livestock density |
| Temperature | | Time series (2000-2013) and spatial variability per UHTU |
| Precipitation | |  |
| Water input | Precipitation |  |
|  | Potential evapotranspiration |  |

- 1. **Data preparation procedure**
     1. Production of plant residues
        1. Cropland residues

To estimate annual plant residue input, we followed Intergovernmental Panel on Climate Change (IPCC) methods. When it was possible, we used the 2006 IPCC method [6]. As this method does not cover all crops, we complemented it with the 1997 IPCC method [7] which, despite being older, encompasses more attributes for a wider array of crops. Table 2 presents the method used per crop and respective parameters. In the 1997 IPCC method [7], crop residue is a fraction of total yield. It is multiplied by the dry matter content, and then converted to carbon content using the carbon fraction in dry matter. In the 2006 IPCC method [6], crop residue (Res) is calculated using the sum of above-ground and below-ground residues. The above-ground residue (AG_DM_) is a function of the total yield. Below-ground residue is a fraction (R_BG-BIO_) of the above-ground residue. Total residues are converted into their carbon content with the carbon fraction in dry matter (C_cont_), according to

|  | $Res=\left( {AG}_{DM}+{{AG}_{DM}\cdot Yield\cdot R}_{BG-BIO} \right)\cdot C_{cont}$, | Eq. (1) |
| --- | --- | --- |

where AG_DM_ for each crop is calculated using

|  | ${AG}_{DM}=Yield\cdot S+Int$, | Eq. (2) |
| --- | --- | --- |

S is the slope and Int is the intersect. S, Int and R_BG-BIO_ were obtained from the 2006 IPCC report [6] (Table 2). These parameters are uncertain and so the IPCC reports a mean and standard deviation for all of them.

Yield data was obtained in FAOSTAT [8] for the period 2004-14. Table 3 presents the full list of the 202 countries covered by FAOSTAT. For each crop in every country, we obtained the mean and standard deviation from the time series. Countries were a part of the definition of UHTUs, so all UHTUs inside a country were assigned the same mean yield and respective standard deviation. As for all variables, in each iteration the yield is selected at random within the distribution established by this mean and standard deviation, and is used to calculate crop residues.

**Table 2.** Parameters from Intergovernmental Panel on Climate Change (IPCC) for crop residue calculation.

|  | **IPCC 1997** | | **IPCC 2006** | | | |
| --- | --- | --- | --- | --- | --- | --- |
| **Crop** | **Dry matter (%)** | **Residue as fraction of yield (dimensionless)** | **Dry matter (%)** | **Slope (S) ±** **2 s.d. (as % of mean) (dimensionless)** | **Intercept (Int) ±** **2 s.d. (as % of mean) (t residues/ha)** | **Ratio of belowground residues to above-ground biomass (R_BG-BIO_) (dimensionless)** |
| Apple | 0.15 | 1 | - | - | - | - |
| Banana | 0.15 | 1 | - | - | - | - |
| Barley | - | - | 0.89 | 0.98 ± 8% | 0.59 ± 41% | 0.22 ± 33% |
| Cabbage | 0.135 | 0.1 | - | - | - | - |
| Carrot | 0.125 | 0.1 | - | - | - | - |
| Cocoa | 0.85 | 1 | - | - | - | - |
| Coconut | 0.15 | 1 | - | - | - | - |
| Coffee | 0.85 | 1 | - | - | - | - |
| Cotton | 0.9 | 1 | - | - | - | - |
| Grapes | 0.15 | 1 | - | - | - | - |
| Groundnut | - | - | 0.95 | 1.07 ± 19% | 1.54 ± 41% | 0.20 ± 50% |
| Maize | - | - | 0.87 | 1.03 ± 3% | 0.61 ± 19% | 0.22 ± 26% |
| Oil palm | 0.15 | 1 | - | - | - | - |
| Olive | 0.15 | 1 | - | - | - | - |
| Onion | 0.1 | 0.1 | - | - | - | - |
| Orange | 0.15 | 1 | - | - | - | - |
| Potato | - | - | 0.22 | 0.10 ± 69% | 1.06 ± 70% | 0.20 ± 50% |
| Rapeseed | - | - | 0.89 | 1.09 ± 2% | 0.88 ± 6% | 0.22 ± 16% |
| Rice | - | - | 0.89 | 0.95 ± 19% | 2.46 ± 41% | 0.16 ± 35% |
| Sorghum | - | - | 0.89 | 0.88 ± 13% | 1.33 ± 27% | 0.22 ± 16% |
| Soybean | - | - | 0.91 | 0.93 ± 31% | 1.35 ± 49% | 0.19 ± 45% |
| Sugarbeet | 0.22 | 0.2 | - | - | - | - |
| Sugarcane | 0.83 | 1 | - | - | - | - |
| Sunflower | 0.933 | 1 | - | - | - | - |
| Sweet potato | - | - | 0.22 | 0.10 ± 69% | 1.06 ± 70% | 0.20 ± 50% |
| Tobacco | 0.15 | 2 | - | - | - | - |
| Tomato | 0.27 | 2 | - | - | - | - |
| Wheat | - | - | 0.89 | 1.61 ± 3% | 0.40 ± 25% | 0.23 ± 41% |

**Table 3.** Full list of countries considered in this study.

| **Country name** | **Country name** |
| --- | --- |
| Afghanistan | Japan |
| Albania | Jordan |
| Algeria | Kazakhstan |
| American Samoa | Kenya |
| Angola | Kiribati |
| Argentina | Korea, Democratic People's Republic of |
| Armenia | Korea, Republic of |
| Australia | Kuwait |
| Austria | Kyrgyzstan |
| Azerbaijan | Lao People's Democratic Republic |
| Bahrain | Latvia |
| Bangladesh | Lebanon |
| Barbados | Lesotho |
| Belarus | Liberia |
| Belize | Lithuania |
| Bhutan | Madagascar |
| Bolivia | Malawi |
| Bosnia and Herzegovina | Malaysia |
| Botswana | Maldives |
| Brazil | Mali |
| British Virgin Islands | Malta |
| Brunei Darussalam | Martinique |
| Bulgaria | Mauritania |
| Burkina Faso | Mauritius |
| Burundi | Mexico |
| Cambodia | Mongolia |
| Cameroon | Montenegro |
| Canada | Montserrat |
| Cape Verde | Morocco |
| Cayman Islands | Mozambique |
| Central African Republic | Nepal |
| Chad | Netherlands |
| Chile | New Caledonia |
| China | New Zealand |
| Colombia | Nicaragua |
| Comoros | Niger |
| Congo | Nigeria |
| Cook Islands | Niue |
| Costa Rica | Norway |
| Cote d'Ivoire | Oman |
| Croatia | Pakistan |
| Cuba | Panama |
| Cyprus | Papua New Guinea |
| Czech Republic | Paraguay |
| Democratic Republic of the Congo | Peru |
| Denmark | Philippines |
| Djibouti | Poland |
| Dominica | Portugal |
| Dominican Republic | Puerto Rico |
| Ecuador | Qatar |
| Egypt | Republic of Moldova |
| El Salvador | Russia |
| Equatorial Guinea | Rwanda |
| Eritrea | Saint Kitts and Nevis |
| Estonia | Saint Lucia |
| Ethiopia | Saint Vincent and the Grenadines |
| Fiji | Samoa |
| Finland | Sao Tome and Principe |
| France | Saudi Arabia |
| French Guiana | Senegal |
| Gabon | Serbia |
| Gambia | Seychelles |
| Georgia | Sierra Leone |
| Ghana | Singapore |
| Greece | Slovakia |
| Grenada | Slovenia |
| Guadeloupe | Solomon Islands |
| Guam | Somalia |
| Guatemala | South Africa |
| Guinea | Spain |
| Guinea-Bissau | Sri Lanka |
| Guyana | Sudan |
| Haiti | Swaziland |
| Honduras | Sweden |
| Hong Kong | Switzerland |
| Hungary | Syrian Arab Republic |
| Iceland | Tajikistan |
| India | Thailand |
| Indonesia | The former Yugoslav Republic of Macedonia |
| Iran (Islamic Republic of) | Timor-Leste |
| Iraq | Togo |
| Ireland | Trinidad and Tobago |
| Israel | Tunisia |
| Italy | Turkey |
| Jamaica | Uganda |
| Turkmenistan | Ukraine |
| Antigua and Barbuda | United Kingdom |
| Bahamas | United Republic of Tanzania |
| Benin | United States |
| Faroe Islands | Uruguay |
| French Polynesia | Uzbekistan |
| Marshall Islands | Venezuela |
| Namibia | Viet Nam |
| Suriname | Wallis and Futuna Islands |
| Tonga | Yemen |
| United Arab Emirates | Zambia |
| Vanuatu | Zimbabwe |
| Western Sahara | Belgium |
| Luxembourg | Taiwan |
| Palestine | Germany |
| Romania | Libyan Arab Jamahiriya |

- - - 1. Forest and grassland residues

We obtained forest residues using the IPCC 2003 [9] method. In transitions to forests there are two distinct periods: (a) while the forest is growing (between plantation and forest climax), and (b) after the forest is mature (from forest climax on). C inputs from forests will vary significantly between the two periods, and will increase as forest grows during period (a), and remain approximately constant after maturity is reached. IPCC 2003 [9] provides transition periods (i.e. the transition time for two broad forest types (broadleaf deciduous and needleleaf evergreen) depending on the climate region (using a classification of thermal zones similar to the one used in this work). IPCC 2003 [9] also provides carbon stocks from residues (above and belowground, litter and dead roots, respectively) during the transition period and for mature forests. This document provides the production of mature forest residues (Res_mature_) and it assumes a linear annual increment in the production of residues (I_res_) between the beginning and end of the growth period (GP) and a null annual increment in production of residues after. Thus, the annual production of residues is equal to the amount of residues produced in the previous period (Res_t-1_) plus I_res_. For example, for broadleaf deciduous forests in “boreal, dry” region, Res_mature_ is 25 t C/ha, GP is 50 years, and annual production of residues during GP (I_res_) is 0.5 t C/ha.year,

|  | $I_{res}= \left\{ \begin{aligned} {{Res}_{mature}}/{GP, if t\leq GP} \\ 0, if t>GP \end{aligned} \right..$ | Eq. (3) |
| --- | --- | --- |
|  | ${Res}_{t}= \left. \begin{aligned} {Res}_{t-1}+I_{res} \end{aligned} \right..$ | Eq. (4) |

Annual forest residues were equally distributed during the twelve months of each year. All IPCC data are on a thermal region and continental basis. Therefore, we also calculated forest residues using this combination. Then, we assigned to all UHTUs in a thermal zone inside each continent the same values. Regarding probability distributions, IPCC 2003 [9] provides the average, minimum and maximum values for each combination of forest type and climate region. We transformed this range of residue values into a normal distribution (obtaining a mean and standard deviation). Then, we used the formulas cited above and propagation of error to calculate means and standard deviations for the forest residues.

The IPCC 2003 [9] method provides not only annual residues for mature forests, but also annual residues during the transition period. The more recent IPCC 2006 [6] also provides a method for the calculation of forest residues, but only for mature forests, which made it unusable in this work. Residues of mature forests from the both methods are similar (Table 1). There are some differences between average value between methods, but the average value of using the IPCC 2006 method [6] is in almost all cases within range. Thus, results for mature forests obtained using both methods would likely be similar in terms of stabilization SOC stock.

**Table 1.** Mature forest residues using IPCC 2003 [9] method and IPCC 2006 method [6], for all forest types.

| **IPCC 2006** | | | | **IPCC 2003** | | | |
| --- | --- | --- | --- | --- | --- | --- | --- |
| **Thermal zone** | **Region** | **Litter production (t C/ha)** | | **Broadleaf Deciduous** | | **Needleleaf Evergreen** | |
|  |  |  |  | **Litter production (t C/ha)** | | | |
|  |  | Average | Standard deviation | Average | Min-Max | Average | Min-Max |
| Tropical rain forest | Africa | 7 | 10 | 2.1 | 1-3 | 5.2 | - |
|  | North America | 6 | 12 |  |  |  |  |
|  | South America | 6 | 8 |  |  |  |  |
|  | Asia | 7 | 15 |  |  |  |  |
|  | Oceania | 8 | 8 |  |  |  |  |
|  | Average | 7 | 5 |  |  |  |  |
| Tropical moist deciduous forest | Africa | 5 | 6 |  |  |  |  |
|  | North America | 4 | 3 |  |  |  |  |
|  | South America | 4 | 3 |  |  |  |  |
|  | Asia | 5 | 14 |  |  |  |  |
|  | Oceania | 5 | 7 |  |  |  |  |
|  | Average | 5 | 4 |  |  |  |  |
| Tropical dry forest | Africa | 3 | 2 |  |  |  |  |
|  | North America | 6 | 7 |  |  |  |  |
|  | South America | 6 | 7 |  |  |  |  |
|  | Asia | 3 | 3 |  |  |  |  |
|  | Oceania | 4 | 3 |  |  |  |  |
|  | Average | 4 | 3 |  |  |  |  |
| Tropical shrubland | Africa | 2 | 5 |  |  |  |  |
|  | North America | 2 | 2 |  |  |  |  |
|  | South America | 2 | 2 |  |  |  |  |
|  | Asia | 2 | 2 |  |  |  |  |
|  | Oceania | 2 | 2 |  |  |  |  |
|  | Average | 2 | 1 |  |  |  |  |
| Tropical mountain systems | Africa | 2 | 4 |  |  |  |  |
|  | North America | 3 | 5 |  |  |  |  |
|  | South America | 3 | 5 |  |  |  |  |
|  | Asia | 3 | 5 |  |  |  |  |
|  | Oceania | 4 | 8 |  |  |  |  |
|  | Average | 3 | 3 |  |  |  |  |
| Subtropical humid forest | North America | 4 | 3 | 3 | 2-3 | 4 | - |
|  | South America | 4 | 3 |  |  |  |  |
|  | Asia | 5 | 14 |  |  |  |  |
|  | Oceania | 5 | 3 |  |  |  |  |
|  | Average | 5 | 4 |  |  |  |  |
| Subtropical dry forest | Africa | 3 | 2 |  |  |  |  |
|  | North America | 6 | 8 |  |  |  |  |
|  | South America | 6 | 8 |  |  |  |  |
|  | Asia | 3 | 3 |  |  |  |  |
|  | Oceania | 3 | 3 |  |  |  |  |
|  | Average | 4 | 3 |  |  |  |  |
| Subtropical steppe | Africa | 2 | 6 |  |  |  |  |
|  | North America | 2 | 2 |  |  |  |  |
|  | South America | 2 | 2 |  |  |  |  |
|  | Asia | 2 | 2 |  |  |  |  |
|  | Oceania | 2 | 2 |  |  |  |  |
|  | Average | 2 | 2 |  |  |  |  |
| Subtropical mountain systems | Africa | 1 | 2 |  |  |  |  |
|  | North America | 3 | 5 |  |  |  |  |
|  | South America | 3 | 5 |  |  |  |  |
|  | Asia | 3 | 5 |  |  |  |  |
|  | Oceania | 4 | 9 |  |  |  |  |
|  | Average | 3 | 3 |  |  |  |  |
| Temperate oceanic forest | Europe | 39 | 11 | 28 | 5-33 | 26 | 5-42 |
|  | North America | 166 | 51 |  |  |  |  |
|  | New Zealand | 71 | 12 |  |  |  |  |
|  | South America | 44 | 10 |  |  |  |  |
|  | Average | 80 | 14 |  |  |  |  |
| Temperate continental forest | Asia | 23 | 7 |  |  |  |  |
|  | Europe | 23 | 7 |  |  |  |  |
|  | North America | 23 | 5 |  |  |  |  |
|  | South America | 23 | 5 |  |  |  |  |
|  | Average | 23 | 6 |  |  |  |  |
| Temperate mountain systems | Asia | 39 | 14 |  |  |  |  |
|  | Europe | 39 | 14 |  |  |  |  |
|  | North America | 25 | 6 |  |  |  |  |
|  | South America | 25 | 6 |  |  |  |  |
|  | Average | 32 | 8 |  |  |  |  |
| Boreal coniferous forest | Asia | 12 | 4 | 25 | 10-58 | 31 | 6-86 |
|  | Europe | 12 | 4 |  |  |  |  |
|  | North America | 12 | 4 |  |  |  |  |
|  | Average | 17 | 3 |  |  |  |  |
| Boreal tundra woodland | Asia | 12 | 5 |  |  |  |  |
|  | Europe | 12 | 5 |  |  |  |  |
|  | North America | 12 | 5 |  |  |  |  |
| Boreal mountain systems | Asia | 19 | 8 |  |  |  |  |
|  | Europe | 19 | 8 |  |  |  |  |
|  | North America | 19 | 8 |  |  |  |  |

For grasslands, the annual production of residues was obtained from Table 6.4 of the 2006 IPCC report [6] - which reports the mean value and error interval. Therefore, in each iteration residues in each UHTU was randomly drawn from the probability distribution.

- - 1. Farmyard manure

Carbon input from farmyard manure application is also a relevant input but there is, to our knowledge, no global database of manure application per crop/land use class. We thus used data from Mueller et al. [10] for total aggregate consumption of nitrogen (N) as an indication of the total regional supplementation of N required by each crop. This total can be provided to plants as synthetic and/or animal-origin organic fertilizer. In the absence of regional parameters for the distribution of the two per land class, we considered three separate scenarios.

In the first scenario, we assumed there was no manure input, meaning that all required N was applied as synthetic fertilizer. In the second, we considered that 50% of the total amount of fertilizer was applied as manure. In that case, an amount of manure equal to 50% of the N requirements was added as an animal-based carbon input. In the third scenario, we considered that the entire amount of fertilizer used was manure (organic fertilizer). We disregarded availability of manure for this calculation. To convert the N content of manure to C, we used the C:N ratio of farmyard manure provided by FAO [11]. In each scenario, the only source of uncertainty considered for the amount of organic fertilizer applied was spatial variability of N consumption. In each UHTU we calculated weighted mean and standard deviation per crop (for standard deviation, we used propagation of error to go from N consumption to manure application). Finally, as with other parameters, in each iteration we randomly selected farmyard manure application.

- - 1. Dung during grazing

In the modified version of RothC used in this work, we took into account ruminant (cattle, sheep and goat) deposition of dung during grazing. We started by determining the stocking rates for each animal type per UHTU. We calculated the number of livestock units (LSU) in grasslands by overlaying global LSU data for each animal type with the UHTU map (given that UHTUs are defined, among other layers, by an LU layer). LSU data were obtained from Gridded Livestock of the World v2.0 [12] (<https://livestock.geo-wiki.org/>). In each UHTU, we obtained the mean LSU and standard deviations. The next step was to multiply LSUs and dung production per animal type and geographical region (PD). Dung N production factors were obtained from the IPCC [6] (table 10.19), and then converted from N to C using the FAO [11] C:N ratio. Finally, the mean dung input per hectare and respective standard deviation were obtained by multiplying the dung production factor (in C) and the LSUs per UHTU using

This procedure is described in the following equation,

|  | ${Dung}_{in, UHTU}= \sum_{animal type} LSU\cdot{PD}_{animal, region}\cdot C/N.$ | Eq. (5) |
| --- | --- | --- |

The IPCC does not report any uncertainty factors for the dung production factor, and so we only considered the spatial uncertainty of the LSU distribution. Table 4 presents the mean carbon input from dung during the grazing per LSU and geographical region.

**Table 4.** Carbon dung during grazing for the three livestock types (t C/LSU).

| **Region** | **Cattle (t C/LSU)** | **Sheep (t C/LSU)** | **Goats (t C/LSU)** | **Total (t C/LSU)** |
| --- | --- | --- | --- | --- |
| North America | 1.12 | 0.10 | 0.09 | 1.31 |
| Western Europe | 1.25 | 0.21 | 0.25 | 1.71 |
| Eastern Europe | 0.84 | 0.22 | 0.25 | 1.32 |
| Oceania | 0.93 | 0.28 | 0.28 | 1.49 |
| Latin America | 0.86 | 0.29 | 0.27 | 1.42 |
| Africa | 0.69 | 0.29 | 0.27 | 1.25 |
| Middle East | 0.80 | 0.29 | 0.27 | 1.36 |
| Asia | 0.80 | 0.29 | 0.27 | 1.36 |

- - 1. Temperature and precipitation

Average monthly precipitation and mean air temperature were both obtained from National Aeronautics and Space Administration’s (NASA) [13,14]. We used data for thirteen years (2000-2013). We calculated mean and standard deviation per month from this time series (e.g. in one UHTU, mean temperature in January is the mean of the average temperature in January for each of the thirteen years) considering the spatial variability within each UHTU (weighted mean of all pixels in each UHTU) and propagation of error. We thus calculated a normal distribution of temperature and precipitation per UHTU. Finally, in each iteration we random selected each climate variable from the respective probability distribution.

- - 1. Water input calculation

To calculate water input per month (W_in,m_), we used the Pfister et al.[15] method, expressed as

|  | $W_{in,m}=P_{m}+I_{m},$ | Eq. (6) |
| --- | --- | --- |
|  | $I_{m}=\left\{ \begin{aligned} {WR}_{m}-P_{m}, if {WR}_{m}>P_{m} \\ 0, otherwise \end{aligned} \right.$, and | Eq. (7) |
|  | ${WR}_{m}=K_{i,m}\cdot{PET}_{m}$, | Eq. (8) |

where $W_{in,m}$ is the sum of monthly precipitation (P_m_) and monthly irrigation (I_m_). Monthly precipitation had already been established (section 1.2.4). Monthly irrigation is the difference between monthly crop water requirement (WR_m_) and P_m_ (if WR_m_ is higher than P_m_). WR_m_ is the multiplication of a crop specific coefficient depending of growth stage (K_i,m_) and monthly potential evapotranspiration (PET_m_). The monthly K_i,m_ were obtained in Chapagain et al. [16] which provides sowing/planting date and duration and K_i,m_ for each stage of crop growth, covering all the crops on a thermal zone basis (Table 5). PET_m_ was obtained using Thornthwaite equation [17], which uses monthly average air temperature, average day length, in hours, and number of days per month obtained in the LP DAAC project [13]. These data covered the period 2000-2013. To obtain mean and standard deviation in all UHTUs, we used the same procedure as for temperature and precipitation (which includes temporal and spatial variability). $W_{in,m}$ mean and standard deviation were obtained using propagation of error, considering as inputs the uncertainty of the parameters R_m_ and PET_m_ (the other variables have no uncertainty reported). Finally, in each iteration we randomly drew $W_{in,m}$ from the respective normal distribution.

**Table 5.** Crop specific coefficient depending of growth stage and respective Planting/Green up date and soil cover period.

|  | **Crop** | **K_i,ini_** | **K_i,mid_** | **K_i,lat_** | **Initial stage** | **Dev. Stage** | **Mid. Stage** | **Late stage** | **Planting / Green up date** | **Soil cover period** |
| --- | --- | --- | --- | --- | --- | --- | --- | --- | --- | --- |
| Tropics | Apple | 0.6 | 0.95 | 0.75 | 60 | 90 | 120 | 95 | 15/jan | All year |
|  | Banana | 1 | 1.2 | 1.1 | 120 | 60 | 180 | 5 | 1/fev | All year |
|  | Barley | 0.3 | 1.15 | 0.25 | 15 | 25 | 50 | 30 | 15/mai | May-Sep |
|  | Cabbage | 0.7 | 1.05 | 0.95 | 40 | 60 | 50 | 15 | 1/jul | Jul-Dec |
|  | Carrot | 0.7 | 1.05 | 0.95 | 20 | 30 | 50 | 20 | 1/dez | Mar-Dec |
|  | Cocoa | 0.9 | 0.95 | 0.95 | 120 | 60 | 180 | 5 | 15/mai | All year |
|  | Coconut | 0.95 | 1 | 1 | 120 | 60 | 180 | 5 | 15/fev | All year |
|  | Coffee | 0.9 | 0.95 | 0.95 | 120 | 60 | 180 | 5 | 15/mai | All year |
|  | Cotton | 0.35 | 1.2 | 0.6 | 30 | 50 | 60 | 55 | 1/jun | Jun-Dec |
|  | Grapes | 0.4 | 0.85 | 0.4 | 20 | 40 | 120 | 60 | 15/abr | Apr-Dec |
|  | Groundnut | 0.4 | 1.15 | 0.6 | 35 | 45 | 35 | 25 | 1/nov | Mar-Nov |
|  | Maize | 0.3 | 1.2 | 0.5 | 20 | 35 | 40 | 30 | 15/jun | Jun-Oct |
|  | Oil palm | 0.9 | 0.95 | 0.95 | 120 | 60 | 180 | 5 | 15/fev | All year |
|  | Olive | 0.65 | 0.7 | 0.7 | 30 | 90 | 60 | 185 | 15/abr | All year |
|  | Onion | 1 | 1 | 0.3 | 20 | 45 | 20 | 10 | 15/out | Jan-Oct |
|  | Orange | 0.7 | 0.65 | 0.7 | 60 | 90 | 120 | 95 | 15/jan | All year |
|  | Potato | 0.5 | 1.15 | 0.75 | 30 | 35 | 50 | 25 | 1/dez | Apr-Dec |
|  | Rapeseed | 0.35 | 1.15 | 0.35 | 30 | 60 | 30 | 30 | 1/dez | Apr-Dec |
|  | Rice | 1.05 | 1.2 | 0.6 | 30 | 30 | 80 | 40 | 15/mai | May-Nov |
|  | Sorghum | 0.3 | 1 | 0.55 | 20 | 35 | 40 | 30 | 1/jun | Jun-Oct |
|  | Soybean | 0.4 | 1.15 | 0.5 | 15 | 15 | 40 | 15 | 1/dez | Feb-Dec |
|  | Sugar beet | 0.35 | 1.2 | 0.7 | 35 | 60 | 70 | 40 | 15/nov | Jun-Nov |
|  | Sugarcane | 0.4 | 1.25 | 0.75 | 30 | 50 | 180 | 60 | 15/mar | Jan-Mar |
|  | Sunflower | 0.35 | 1.15 | 0.35 | 25 | 35 | 45 | 25 | 15/nov | Mar-Nov |
|  | Sweet potato | 0.5 | 1.15 | 0.65 | 15 | 30 | 50 | 30 | 1/out | Feb-Oct |
|  | Tobacco | 0.5 | 1.15 | 0.8 | 20 | 30 | 30 | 30 | 15/fev | Feb-Jun |
|  | Tomato | 0.6 | 1.15 | 0.8 | 30 | 40 | 40 | 25 | 15/jan | Jan-May |
|  | Wheat | 0.3 | 1.15 | 0.3 | 15 | 25 | 50 | 30 | 15/mai | May-Sep |
| Subtropics summer rainfall | Apple | 0.6 | 0.95 | 0.75 | 60 | 90 | 120 | 95 | 15/jan | All year |
|  | Banana | 1 | 1.2 | 1.1 | 120 | 60 | 180 | 5 | 1/fev | All year |
|  | Barley | 0.3 | 1.15 | 0.25 | 15 | 25 | 50 | 30 | 15/mai | May-Sep |
|  | Cabbage | 0.7 | 1.05 | 0.95 | 40 | 60 | 50 | 15 | 1/nov | Apr-Nov |
|  | Carrot | 0.7 | 1.05 | 0.95 | 20 | 30 | 50 | 20 | 1/dez | Mar-Dec |
|  | Cocoa | 0.9 | 0.95 | 0.95 | 120 | 60 | 180 | 5 | 15/mai | All year |
|  | Coconut | 0.95 | 1 | 1 | 120 | 60 | 180 | 5 | 15/fev | All year |
|  | Coffee | 0.9 | 0.95 | 0.95 | 120 | 60 | 180 | 5 | 15/mai | All year |
|  | Cotton | 0.35 | 1.2 | 0.6 | 30 | 50 | 60 | 55 | 1/jun | Jun-Dec |
|  | Grapes | 0.4 | 0.85 | 0.4 | 20 | 40 | 120 | 60 | 15/abr | Apr-Dec |
|  | Groundnut | 0.4 | 1.15 | 0.6 | 35 | 45 | 35 | 25 | 1/jul | Jul-Nov |
|  | Maize | 0.3 | 1.2 | 0.5 | 20 | 35 | 40 | 30 | 15/jun | Jun-Oct |
|  | Oil palm | 0.9 | 0.95 | 0.95 | 120 | 60 | 180 | 5 | 15/fev | All year |
|  | Olive | 0.65 | 0.7 | 0.7 | 30 | 90 | 60 | 185 | 15/abr | All year |
|  | Onion | 1 | 1 | 0.3 | 20 | 45 | 20 | 10 | 15/out | Jan-Oct |
|  | Orange | 0.7 | 0.65 | 0.7 | 60 | 90 | 120 | 95 | 15/jan | All year |
|  | Potato | 0.5 | 1.15 | 0.75 | 25 | 30 | 45 | 30 | 1/jul | Jul-Nov |
|  | Rapeseed | 0.35 | 1.15 | 0.35 | 30 | 60 | 30 | 30 | 1/out | Feb-Oct |
|  | Rice | 1.05 | 1.2 | 0.6 | 30 | 30 | 60 | 30 | 15/jun | Jun-Nov |
|  | Sorghum | 0.3 | 1 | 0.55 | 20 | 35 | 40 | 30 | 1/jun | Jun-Oct |
|  | Soybean | 0.4 | 1.15 | 0.5 | 20 | 30 | 60 | 25 | 1/jun | Jun-Oct |
|  | Sugar beet | 0.35 | 1.2 | 0.7 | 45 | 75 | 80 | 30 | 15/nov | Jul-Nov |
|  | Sugarcane | 0.4 | 1.25 | 0.75 | 30 | 50 | 180 | 60 | 15/mar | Jan-Mar |
|  | Sunflower | 0.35 | 1.15 | 0.35 | 25 | 35 | 45 | 25 | 15/nov | Mar-Nov |
|  | Sweet potato | 0.5 | 1.15 | 0.65 | 15 | 30 | 50 | 30 | 1/out | Feb-Oct |
|  | Tobacco | 0.5 | 1.15 | 0.8 | 20 | 30 | 30 | 30 | 15/mai | May-Sep |
|  | Tomato | 0.6 | 1.15 | 0.8 | 30 | 40 | 40 | 25 | 15/jan | Jan-May |
|  | Wheat | 0.7 | 1.15 | 0.3 | 15 | 25 | 50 | 30 | 15/mai | May-Sep |
| Subtropics winter rainfall | Apple | 0.6 | 0.95 | 0.75 | 60 | 90 | 120 | 95 | 15/jan | All year |
|  | Banana | 1 | 1.2 | 1.1 | 120 | 60 | 180 | 5 | 1/fev | All year |
|  | Barley | 0.3 | 1.15 | 0.25 | 20 | 60 | 70 | 30 | 15/nov | May-Nov |
|  | Cabbage | 0.7 | 1.05 | 0.95 | 40 | 60 | 50 | 15 | 1/jul | Jul-Dec |
|  | Carrot | 0.7 | 1.05 | 0.95 | 30 | 40 | 60 | 20 | 15/mar | Mar-Aug |
|  | Cocoa | 0.9 | 0.95 | 0.95 | 120 | 60 | 180 | 5 | 15/mai | All year |
|  | Coconut | 0.95 | 1 | 1 | 120 | 60 | 180 | 5 | 15/fev | All year |
|  | Coffee | 0.9 | 0.95 | 0.95 | 120 | 60 | 180 | 5 | 15/mai | All year |
|  | Cotton | 0.35 | 1.2 | 0.6 | 30 | 50 | 60 | 55 | 1/jul | Jan-Jul |
|  | Grapes | 0.4 | 0.85 | 0.4 | 20 | 40 | 120 | 60 | 15/abr | Apr-Dec |
|  | Groundnut | 0.4 | 1.15 | 0.6 | 35 | 45 | 35 | 25 | 1/jun | Jun-Oct |
|  | Maize | 0.3 | 1.2 | 0.5 | 30 | 40 | 50 | 30 | 15/abr | Apr-Sep |
|  | Oil palm | 0.9 | 0.95 | 0.95 | 120 | 60 | 180 | 5 | 15/fev | All year |
|  | Olive | 0.65 | 0.7 | 0.7 | 30 | 90 | 60 | 185 | 15/abr | All year |
|  | Onion | 1 | 1 | 0.3 | 20 | 45 | 20 | 10 | 15/out | Jan-Oct |
|  | Orange | 0.7 | 0.65 | 0.7 | 60 | 90 | 120 | 95 | 15/jan | All year |
|  | Potato | 0.5 | 1.15 | 0.75 | 25 | 30 | 45 | 30 | 15/mar | Mar-Jul |
|  | Rapeseed | 0.35 | 1.15 | 0.35 | 30 | 60 | 30 | 30 | 1/jun | Jun-Oct |
|  | Rice | 1.05 | 1.2 | 0.6 | 30 | 30 | 80 | 40 | 1/jun | Jun-Nov |
|  | Sorghum | 0.3 | 1 | 0.55 | 20 | 35 | 40 | 30 | 1/jul | Jul-Nov |
|  | Soybean | 0.4 | 1.15 | 0.5 | 20 | 30 | 60 | 25 | 15/abr | Apr-Aug |
|  | Sugar beet | 0.35 | 1.2 | 0.7 | 50 | 40 | 50 | 40 | 15/abr | Apr-Oct |
|  | Sugarcane | 0.4 | 1.25 | 0.75 | 30 | 50 | 180 | 60 | 15/mar | Jan-Mar |
|  | Sunflower | 0.35 | 1.15 | 0.35 | 25 | 35 | 45 | 25 | 15/nov | Mar-Nov |
|  | Sweet potato | 0.5 | 1.15 | 0.65 | 20 | 30 | 60 | 40 | 1/abr | Apr-Aug |
|  | Tobacco | 0.5 | 1.15 | 0.8 | 20 | 30 | 30 | 30 | 15/fev | Feb-Jun |
|  | Tomato | 0.6 | 1.15 | 0.8 | 30 | 40 | 40 | 25 | 15/jan | Jan-May |
|  | Wheat | 0.7 | 1.15 | 0.3 | 20 | 60 | 70 | 30 | 15/nov | May-Nov |
| Oceanic temperate | Apple | 0.6 | 0.95 | 0.75 | 60 | 90 | 120 | 95 | 15/jan | All year |
|  | Banana | 0.5 | 0.85 | 0.6 | 30 | 45 | 35 | 10 | 1/mai | May-Aug |
|  | Barley | 0.3 | 1.15 | 0.25 | 40 | 60 | 60 | 40 | 15/nov | Jun-Nov |
|  | Cabbage | 0.7 | 1.05 | 0.95 | 40 | 60 | 50 | 15 | 1/mai | May-Oct |
|  | Carrot | 0.4 | 0.85 | 0.75 | 20 | 40 | 120 | 60 | 1/jun | Jan-Jun |
|  | Cocoa | 0.9 | 0.95 | 0.95 | 120 | 60 | 180 | 5 | 15/mai | All year |
|  | Coconut | 0.35 | 1.2 | 0.6 | 30 | 50 | 60 | 55 | 15/mai | May-Nov |
|  | Coffee | 0.9 | 0.95 | 0.95 | 120 | 60 | 180 | 5 | 15/mai | All year |
|  | Cotton | 0.7 | 1 | 0.7 | 20 | 35 | 110 | 45 | 15/Sep | Apr-Sep |
|  | Grapes | 0.9 | 0.95 | 0.95 | 120 | 60 | 180 | 5 | 15/mai | All year |
|  | Groundnut | 0.4 | 1.05 | 0.75 | 25 | 35 | 40 | 30 | 15/Aug | Aug-Dec |
|  | Maize | 0.3 | 1 | 0.3 | 20 | 30 | 55 | 35 | 15/mai | May-Oct |
|  | Oil palm | 0.35 | 1.1 | 0.25 | 25 | 35 | 50 | 40 | 15/abr | Apr-Sep |
|  | Olive | 0.7 | 1.05 | 0.95 | 40 | 60 | 50 | 15 | 1/mai | May-Oct |
|  | Onion | 0.6 | 0.95 | 0.75 | 60 | 90 | 120 | 95 | 15/jan | All year |
|  | Orange | 0.5 | 0.3 | 0.3 | 60 | 120 | 175 | 10 | 15/jan | All year |
|  | Potato | 0.5 | 1.1 | 0.95 | 25 | 30 | 25 | 10 | 1/mar | Mar-May |
|  | Rapeseed | 0.35 | 1.15 | 0.35 | 30 | 60 | 60 | 30 | 1/mai | May-Oct |
|  | Rice | 0.3 | 1.15 | 0.25 | 160 | 75 | 75 | 25 | 15/out | Sep-Oct |
|  | Sorghum | 0.3 | 1.15 | 0.3 | 30 | 30 | 40 | 30 | 15/out | Feb-Oct |
|  | Soybean | 0.7 | 0.9 | 0.85 | 10 | 15 | 20 | 10 | 1/mai | May-Jun |
|  | Sugar beet | 0.5 | 1.15 | 1.1 | 20 | 30 | 30 | 20 | 15/abr | Apr-Jul |
|  | Sugarcane | 0.4 | 1.1 | 0.3 | 20 | 30 | 60 | 40 | 1/mai | May-Sep |
|  | Sunflower | 0.7 | 1 | 0.95 | 35 | 50 | 45 | 10 | 15/fev | Feb-Jul |
|  | Sweet potato | 0.4 | 1.25 | 0.75 | 30 | 60 | 180 | 95 | 15/mar | All year |
|  | Tobacco | 0.5 | 1.15 | 0.8 | 20 | 30 | 30 | 30 | 15/mai | May-Sep |
|  | Tomato | 0.7 | 1.05 | 0.95 | 35 | 45 | 40 | 15 | 1/mai | May-Sep |
|  | Wheat | 0.7 | 1.15 | 0.3 | 160 | 75 | 75 | 25 | 15/out | Sep-Oct |
| Sub-continental temperate and continental temperate | Apple | 0.6 | 0.95 | 0.75 | 60 | 90 | 120 | 95 | 15/jan | All year |
|  | Banana | 0.5 | 0.85 | 0.6 | 30 | 45 | 35 | 10 | 1/mai | May-Aug |
|  | Barley | 0.3 | 1.15 | 0.25 | 40 | 30 | 40 | 20 | 15/out | Feb-Oct |
|  | Cabbage | 0.7 | 1.05 | 0.95 | 40 | 60 | 50 | 15 | 15/mai | May-Oct |
|  | Carrot | 0.4 | 0.85 | 0.75 | 20 | 40 | 120 | 60 | 1/jun | Jan-Jun |
|  | Cocoa | 0.9 | 0.95 | 0.95 | 120 | 60 | 180 | 5 | 15/mai | All year |
|  | Coconut | 0.35 | 1.2 | 0.6 | 30 | 50 | 60 | 55 | 15/mai | May-Nov |
|  | Coffee | 0.9 | 0.95 | 0.95 | 120 | 60 | 180 | 5 | 15/mai | All year |
|  | Cotton | 0.7 | 1 | 0.7 | 20 | 35 | 110 | 45 | 15/jan | Jan-Aug |
|  | Grapes | 0.9 | 0.95 | 0.95 | 120 | 60 | 180 | 5 | 15/mai | All year |
|  | Groundnut | 0.4 | 1.05 | 0.75 | 25 | 35 | 40 | 30 | 15/Aug | Aug-Dec |
|  | Maize | 0.3 | 1 | 0.3 | 20 | 30 | 55 | 35 | 15/mai | May-Oct |
|  | Oil palm | 0.35 | 1.1 | 0.25 | 25 | 35 | 50 | 40 | 15/abr | Apr-Sep |
|  | Olive | 0.7 | 1.05 | 0.95 | 40 | 60 | 50 | 15 | 15/mai | May-Oct |
|  | Onion | 0.6 | 0.95 | 0.75 | 60 | 90 | 120 | 95 | 15/jan | All year |
|  | Orange | 0.5 | 0.3 | 0.3 | 60 | 120 | 175 | 10 | 15/jan | All year |
|  | Potato | 0.5 | 1.1 | 0.95 | 25 | 30 | 25 | 10 | 1/mar | Mar-May |
|  | Rapeseed | 0.35 | 1.15 | 0.35 | 30 | 60 | 60 | 30 | 15/Sep | Mar-Sep |
|  | Rice | 0.3 | 1.15 | 0.25 | 20 | 60 | 70 | 30 | 1/dez | May-Dec |
|  | Sorghum | 0.3 | 1.15 | 0.3 | 30 | 30 | 40 | 30 | 1/dez | Apr-Dec |
|  | Soybean | 0.7 | 0.9 | 0.85 | 10 | 15 | 20 | 10 | 1/mai | May-Jun |
|  | Sugar beet | 0.5 | 1.15 | 1.1 | 20 | 30 | 30 | 20 | 15/abr | Apr-Jul |
|  | Sugarcane | 0.4 | 1.1 | 0.3 | 20 | 30 | 60 | 40 | 1/mai | May-Sep |
|  | Sunflower | 0.7 | 1 | 0.95 | 35 | 50 | 45 | 10 | 15/fev | Feb-Jul |
|  | Sweet potato | 0.4 | 1.25 | 0.75 | 30 | 60 | 180 | 95 | 15/mar | All year |
|  | Tobacco | 0.5 | 1.15 | 0.8 | 20 | 30 | 30 | 30 | 15/mai | May-Sep |
|  | Tomato | 0.7 | 1.05 | 0.95 | 35 | 45 | 40 | 15 | 15/mai | May-Sep |
|  | Wheat | 0.7 | 1.15 | 0.3 | 20 | 60 | 70 | 30 | 1/dez | May-Dec |
| Sub-continental boreal, continental boreal and polar/arctic | Apple | 0.6 | 0.95 | 0.75 | 60 | 90 | 120 | 95 | 15/jan | All year |
|  | Banana | 0.5 | 0.85 | 0.6 | 30 | 45 | 35 | 10 | 1/mai | All year |
|  | Barley | 0.3 | 1.15 | 0.25 | 160 | 75 | 75 | 25 | 15/out | May-Sep |
|  | Cabbage | 0.7 | 1.05 | 0.95 | 40 | 60 | 50 | 15 | 1/mai | Jul-Dec |
|  | Carrot | 0.4 | 0.85 | 0.75 | 20 | 40 | 120 | 60 | 1/jun | Mar-Dec |
|  | Cocoa | 0.9 | 0.95 | 0.95 | 120 | 60 | 180 | 5 | 15/mai | All year |
|  | Coconut | 0.35 | 1.2 | 0.6 | 45 | 90 | 45 | 45 | 15/mar | All year |
|  | Coffee | 0.9 | 0.95 | 0.95 | 120 | 60 | 180 | 5 | 15/mai | All year |
|  | Cotton | 0.7 | 1 | 0.7 | 20 | 35 | 110 | 45 | 15/jan | Jun-Dec |
|  | Grapes | 0.9 | 0.95 | 0.95 | 120 | 60 | 180 | 5 | 15/mai | Apr-Dec |
|  | Groundnut | 0.4 | 1.05 | 0.75 | 25 | 35 | 40 | 30 | 15/Aug | Mar-Nov |
|  | Maize | 0.3 | 1 | 0.3 | 20 | 30 | 55 | 35 | 15/mai | Jun-Oct |
|  | Oil palm | 0.35 | 1.1 | 0.25 | 25 | 35 | 50 | 40 | 15/abr | All year |
|  | Olive | 0.7 | 1.05 | 0.95 | 40 | 60 | 50 | 15 | 1/mai | All year |
|  | Onion | 0.6 | 0.95 | 0.75 | 60 | 90 | 120 | 95 | 15/jan | Jan-Oct |
|  | Orange | 0.5 | 0.3 | 0.3 | 60 | 120 | 175 | 10 | 15/jan | All year |
|  | Potato | 0.5 | 1.1 | 0.95 | 25 | 30 | 25 | 10 | 1/mar | Apr-Dec |
|  | Rapeseed | 0.35 | 1.15 | 0.35 | 30 | 60 | 60 | 30 | 15/mai | Apr-Dec |
|  | Rice | 0.3 | 1.15 | 0.25 | 160 | 75 | 75 | 25 | 15/nov | May-Nov |
|  | Sorghum | 0.3 | 1.15 | 0.3 | 30 | 30 | 40 | 30 | 15/nov | Jun-Oct |
|  | Soybean | 0.7 | 0.9 | 0.85 | 10 | 15 | 20 | 10 | 1/mai | Feb-Dec |
|  | Sugar beet | 0.5 | 1.15 | 1.1 | 20 | 30 | 30 | 20 | 15/abr | Jun-Nov |
|  | Sugarcane | 0.4 | 1.1 | 0.3 | 20 | 30 | 60 | 40 | 1/mai | Jan-Mar |
|  | Sunflower | 0.7 | 1 | 0.95 | 35 | 50 | 45 | 10 | 15/fev | Mar-Nov |
|  | Sweet potato | 0.4 | 1.25 | 0.75 | 30 | 60 | 180 | 95 | 15/mar | Feb-Oct |
|  | Tobacco | 0.5 | 1.15 | 0.8 | 20 | 30 | 30 | 30 | 15/mai | Feb-Jun |
|  | Tomato | 0.7 | 1.05 | 0.95 | 35 | 45 | 40 | 15 | 1/mai | Jan-May |
|  | Wheat | 0.4 | 1.15 | 0.3 | 160 | 75 | 75 | 25 | 15/nov | May-Sep |

- 1. **Definition of unique homogeneous territorial units**

UHTUs were defined as a geographical combination of five layers, namely thermal zones, land cover, soil type, soil texture and country. Thermal zone data was obtained from the GAEZ [18], which divides the world into 12 zones. We excluded arctic and desert regions. We used data from the Land Processes Distributed Active Archive Center (LP DAAC) [19] for attribution of land classes. This source considers 16 LC classes, out of which we excluded “water” and “unclassified” regions from the analysis. Soil type and texture data were obtained from the World Reference Base (WRB), as depicted in Fischer et al. [20]. We considered the 8 types of soil and 13 types of texture from WRB (clay (heavy); silty clay; clay; silty clay loam; clay loam; silt; silt loam; sandy clay; loam; sandy clay loam; sandy loam; loamy sand; and sand). We obtained the country borders from the World Borders Dataset [21].

- 1. **Model initialization**

RothC divides SOC stock into 5 pools: inert organic matter (IOM), easily decomposable plant material (DPM), resistant plant material (RPM), microbial biomass (BIO) and humified organic matter (HUM). The RothC manual [22] recommends the use of a DPM/RPM ratio of 1.44 for agricultural crops and grasslands. For permanent crops, the DPM/RPM ratio is 1, due to the greater content of lignin in permanent crop soils, which has higher resistance to decomposition [23]. For forests, the DPM/RPM ratio is 0.25. Farmyard manure inputs have a specific distribution for each pool. The distributions is: 49% to DPM, 48% to RPM and 2% to humus [22]. Then, DPM and RPM inputs are distributed among the BIO and HUM pools according to fixed ratios. The IOM pool does not change. The initial SOC stock obtained from European Soil Data Centre (ESDAC) [24] is an aggregated measure, meaning that it is not divided between these pools. In order to initialize the model and distribute initial SOC by the corresponding compartments, we used the method by Weihermüller and colleagues [25]

|  | $IOM=0.049\cdot{SOC}^{1.139}$, | Eq. (9) |
| --- | --- | --- |
|  | $RPM=\left( 0.1847\cdot SOC+0.1555 \right)\times{(Clay+1.2750)}^{-0.1158}$, | Eq. (10) |
|  | $HUM=\left( 0.7148 \cdot SOC+0.5069 \right)\times{(Clay+0.3421)}^{0.0184}$, | Eq. (11) |
|  | $BIO=\left( 0.0140 \cdot SOC+0.0075 \right)\times{(Clay+8.8473)}^{0.0567}$, | Eq. (12) |

where SOC is soil organic carbon stock (t C/ha), Clay is the clay content expressed in %, IOM is the inert organic matter (t C/ha), RPM is the resistant plant material (t C/ha), BIO is the microbial biomass (t C/ha), HUM is the humified organic matter (t C/ha), and SOC is soil organic carbon (t C/ha). These distributions only depend on the initial SOC values and clay fraction of the soil. Thus, the DPM pool is calculated as the difference between initial SOC and the sum of the results of equations Eq. (9)-Eq. (12) (if the sum is less than total SOC stock). If sum of the four pools is equal to the SOC stock (by construction can be higher than total SOC stock), then the DPM pool is an empty pool.

RothC calculates the decay rate, and consequently the mineralization rate, for each pool in every time step (in our work: one month). First, fresh SOC inputs are distributed between two pools: DPM and RPM. The partition between these two pools (DPM/RPM ratio) is a predetermined parameter depending on the LU class.

- 1. **Additional methods for supplementary results**

The following methods describe additional analyses performed on the results, which were mostly omitted from the main paper. The results of these analyses are presented in the present supporting materials file (section 2).

- - 1. **Comparison between LU classes**

To assess if the naturalized LU classes (i.e. forests and grassland) always achieve higher stabilization SOC stock when compared with artificial LU classes (i.e. croplands), we subtracted, on an UHTU basis, mean stabilization SOC stocks of naturalized LU classes from mean stabilization SOC stocks of artificial LU classes (in the UHTUs where both LU classes coexist). Here, we also assessed the influence of management practices by subtracting, for the same crop and UHTU, the SOC stocks for two classes, (e.g. the maintenance residues on the field increases stabilization SOC stock for cereals classes by 12 t C/ha).

- - 1. **Validation using LUCAS data**

We compared our results (stabilization SOC stocks) with data collected during the LUCAS Project [26] in Europe using Pearson’s correlation coefficients between comparable LU classes. To enable the comparison, we produced a correspondence key between our LU classes classification and LUCAS Project [26] classification (Table 6). For croplands the correspondence key was not required, as there was a direct match. However, we considered multiple management practices (irrigation/rainfed and residues left/removed on/from the field). For these cases, we use all possibilities, e.g. irrigated maize and rainfed maize were compared with same maize class from LUCAS Project [26]. We used software IBM SPSS version 25 to perform Pearson’s correlation tests.

**Table 6.** Correspondence between land use classification used in this study and land use classifications from LUCAS Project [26].

| **Crop name (this study)** | **Crop name (LUCAS Project)** |
| --- | --- |
| Apple | Apple |
| Cotton | Cotton |
| Grape | Grape |
| Maize | Maize |
| Olive | Olive |
| Orange | Orange |
| Potato | Potato |
| Rice | Rice |
| Soybean | Soybean |
| Sugar beet | Sugar beet |
| Sunflower | Sunflower |
| Tobacco | Tobacco |
| Tomato | Tomato |
| Wheat | Wheat (common) |
| Boreal coniferous forest | Coniferous woodland |
| Boreal mountain system |  |
| Temperate continental forest | Mixed woodland |
| Temperate oceanic forest |  |
| Boreal tundra woodland | Shrubland without tree cover |
| Grassland | Grassland with sparse tree/shrub cover |
|  | Grassland without tree/shrub cover |
|  | Spontaneously vegetated surfaces |

- - 1. **Sensitivity to input parameters**

RothC involves multiple parameters and its interpretation entails difficulties tracing how each input influences results. Thus, we used a linear regression approach to estimate how the main output, SOC stock changes with the input parameters. This exercise allowed us to obtain insights on the contribution of individual variables to the results. The dependent variable of the linear approximation was the difference between stabilization SOC stock and in the baseline year, i.e. the SOC difference between the last year simulated and the initial value (∆SOC). The independent variables were the inputs of the model: mean monthly temperature (T), monthly precipitation (P), crop carbon residues (Res), initial SOC (SOC_LU1_) and farmyard manure (FMY), as shown in

|  | $SOC\left[ t C.{ha}^{-1} \right]=\Delta SOC{\times\alpha}_{0}+T{\times\alpha}_{1}+P{\times\alpha}_{2}+Res\times\alpha_{3}+{SOC}_{ini}{\times\alpha}_{4}+C{\times\alpha}_{5}+FYM{\times\alpha}_{6}+\varepsilon$. | Eq. (13) |
| --- | --- | --- |

The ε corresponds to the variance unaccounted for by the model. We estimated the linear regression for all LU classes in each thermal zone basis using only the mean values, e.g. the average monthly temperature in the Temperate, continental thermal zone is the average for all UHTUs in this region. After estimating the regression coefficients *α_0_* through *α_6_*, we plotted the dependent variable as a function of each independent variable separately setting all other variables equal to their mean in the 100 iterations.

1. **Supplementary results**
   1. **Comparison between LU**
      1. Natural LU versus artificial LU

In almost every UHTUs, forests reach higher stabilization SOC stock than croplands. As an example, Figure 1 depicts the comparison between boreal coniferous forest with maize and wheat (with different management practices). In green are represented areas where boreal coniferous forest reach higher stabilization SOC stock than maize and wheat (in red, the opposite case is represented). There are some notable exceptions to the general rule that forests accumulate more SOC in some regions for agricultural classes with high plant residues such as irrigated wheat (visible in red in Fig. 1-e and Fig. 1-g). Grasslands also typically accumulate more SOC than croplands, but with more exceptions than forests (Fig. 2 in the main text). Grasslands in some cases may accumulate more SOC than forests, when plant residues are lower in forests in a given region than grasslands residue input. Nevertheless, overall a greater portion of forest residues is resistant material that is not decomposable, while SOC accumulation in grasslands is more labile[1]. RothC takes this into account in the DPM/RPM ratio, which is 0.25 for forests (20% is DPM and 80% is RPM) and for grasslands it is 1.44 (59% is DPM and 41% is RPM).

Nevertheless, as mentioned in the main text, there are also other croplands which reach higher stabilization SOC stocks than naturalized LU classes, e.g. tomato in western Scandinavia. Tomato, as high-yield maize and wheat in some locations, has considerably high crop residue production.

**
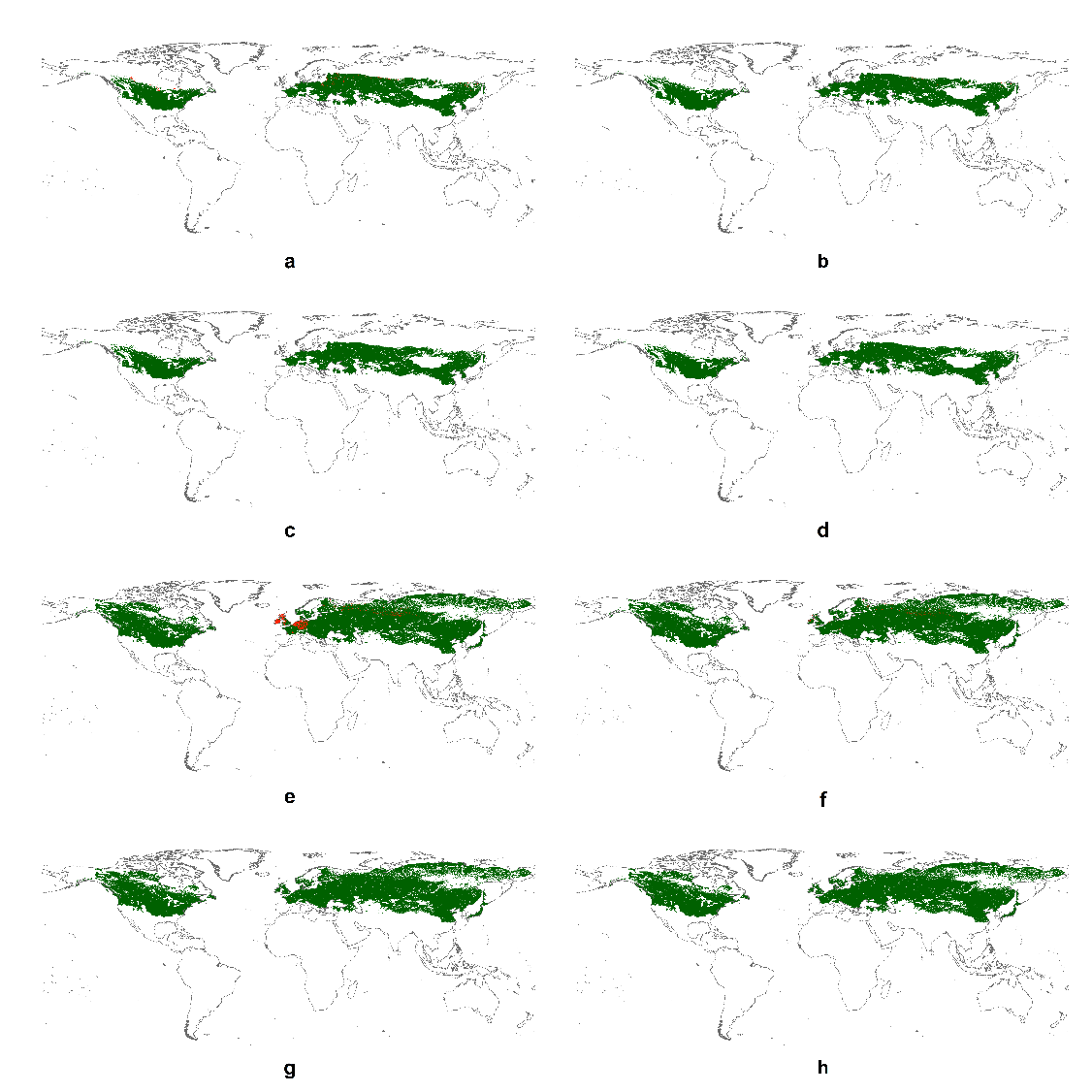
**

**Figure 1.** Visual representation of the regions where soil organic carbon (SOC) stock (t C/ha) is higher for boreal coniferous forest than agricultural land uses (in green) or lower (in red). Agricultural land uses and practices represented: a, b, c, d – maize, e, f, g, h – wheat; a, c, e, g – irrigated, b, d, f, h – rainfed; a, b, e, f - residues left on the field, c, d, g, h - residues removed from the field.

- 1. **Validation with LUCAS data**

The full list of correlations between stabilization SOC stocks and LUCAS Project[26] field measurements is in Table S2 in the “S2. Correlation analysis” file. As mentioned in the main text, correlations are low but significant. Grasslands are the LU class with highest mean correlation, 0.28 (min: 0.23; max: 0.33). For forests the correlation factor is 0.26 (min: -0.24; max: 0.99), and for croplands it is 0.15 (min: 0; max: 1,00). Within the croplands class, irrigated crops are better correlated than rainfed crops, at 0.16 and 0.12, respectively. Further, cereal crops are slightly better correlated with LUCAS data (0.17) than the other crop groups (0.14).

The reason why the correlation between SOC stocks obtained here and measured during LUCAS is lower for croplands could be that there is no assurance that SOC at each LUCAS location was stabilized (recent transformations), or that the site may actually harbour a crop rotation system (while our results are for a single crop). In naturalized LU classes the probability of finding rotations is lower, and so correlations are higher. Differences may be, for forests, due to the fact that we were unable to model agri-forestry systems that are within the LUCAS sample sites. For grasslands, the lack of a stronger correlation may be because of extreme variability in grassland composition throughout Europe, while due to lack of data we have only one grassland class. One transversal reason why the correlations, despite significant, are relatively low is that LUCAS data considers 20 cm depth and our results are for 30 cm.

- 1. **Sensitivity of the model to input parameters**

Carbon in plant residues is the critical model input, i.e. it is the main cause for the differences between final and initial SOC, regardless of LU class. Plant residues are also the input that ranges more widely between LU classes. Fig. 2 shows the results of the analysis performed using the regression model, indicating the relationship between the SOC differential and the inputs parameters of RothC for “maize irrigated, residues left on the field”, in six thermal zones (a, tropics, lowland; b, subtropics; c, summer rainfall; d, subtropics, winter rainfall; d, temperate, oceanic; e, temperate, sub-continental; and f, boreal, continental).

The horizontal axis is in a relative scale, where the variable value is divided by the respective mean value. The R^2^ of the regressions is relatively low (0.162 to 0.413) because they linearize effects of the independents, while RothC is an exponential model.

Fig. 2 shows that the SOC change is mostly sensitive to changes in carbon residue (highest slope), regardless of thermal zone. Fig. 2 shows also that the effect of initial SOC and temperature depends on the thermal zone. Higher water input (precipitation and irrigation) typically leads to a SOC loss except in the “temperate, oceanic” thermal zone. Water availability in soils is usually a cause for increased bacterial activity and higher SOC mineralization rates without compensation in C input due to higher ecosystem productivity. The temperate oceanic region has lower precipitation water inputs, and consequently water increase through irrigation can spark high yields that more than compensate for higher mineralization. Air temperature has similar effects on yield and mineralization. Initial SOC stocks have a negative effect in the variation of SOC simply due to the construction of the dependent variable (difference between stabilization and initial SOC). The effect of clay is almost null for all thermal zones. Results for the scenario where fertilization is from organic manure (i.e. manure input is considered) are similar due the fact that manure input contribute less than residues to SOC changes.


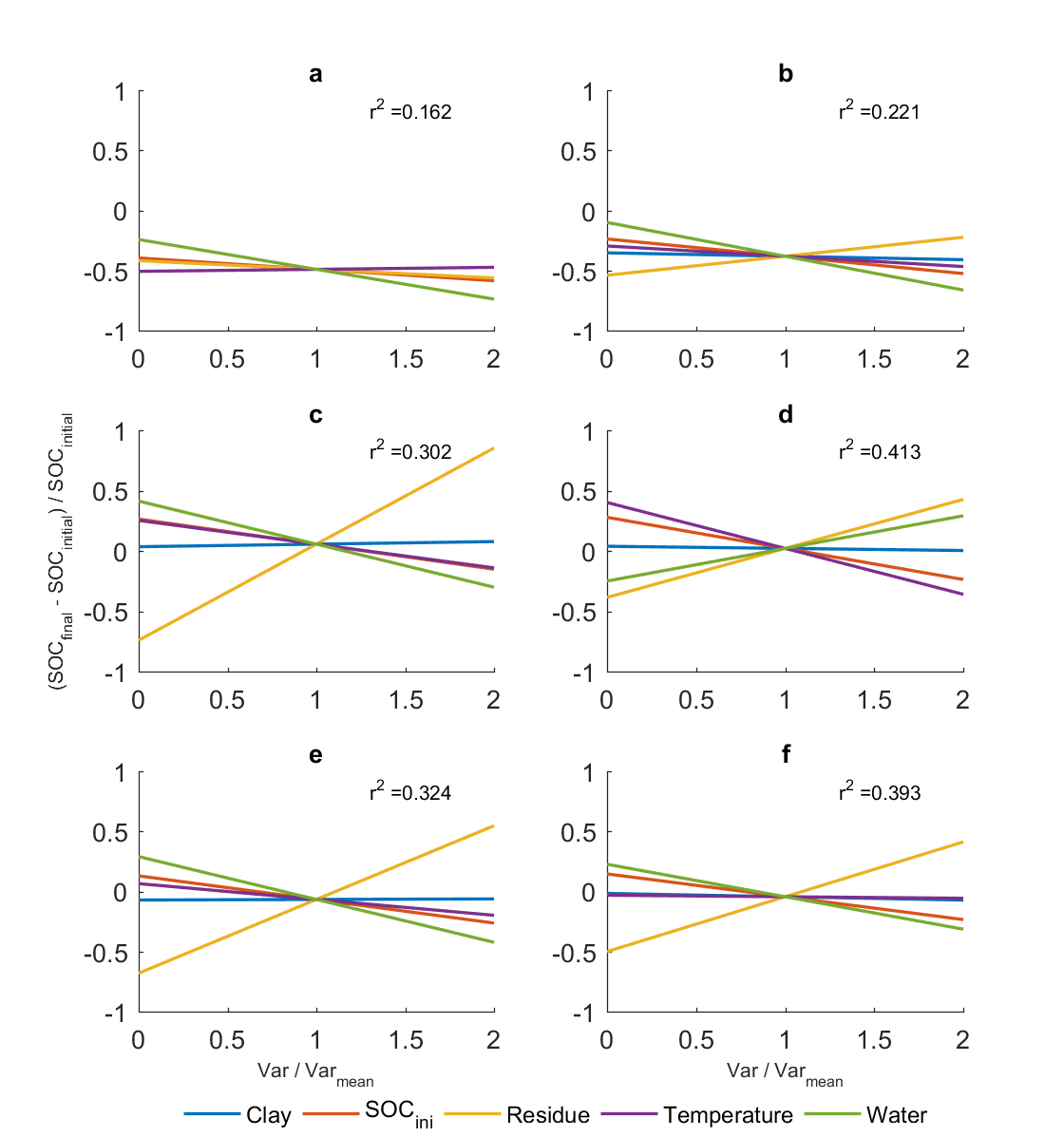


**Figure 2.** Linear relationship between Soil Organic Carbon (SOC) stock content differential from start to finish of the RothC simulations and the main input variables of the model. a, Tropics, lowland. b, Subtropics, summer rainfall. c, Subtropics, winter rainfall. d, Temperate, oceanic. e, Temperate, sub-continental. f, Temperate, continental.

**References**

1. Coleman, K.; Jenkinson, D. S.; Crocker, G. J.; Grace, P. R.; Klír, J.; Körschens, M.; Poulton, P. R.; Richter, D. D. Simulating trends in soil organic carbon in long-term experiments using RothC-26.3. *Geoderma* **1997**, *81*, 29–44, doi:10.1016/S0016-7061(97)00079-7.

2. Hashimoto, S.; Wattenbach, M.; Smith, P. Litter carbon inputs to the mineral soil of Japanese Brown forest soils: Comparing estimates from the RothC model with estimates from MODIS. *J. For. Res.* **2011**, *16*, 16–25, doi:10.1007/s10310-010-0209-6.

3. Xu, X.; Liu, W.; Kiely, G. Modeling the change in soil organic carbon of grassland in response to climate change: Effects of measured versus modelled carbon pools for initializing the Rothamsted Carbon model. *Agric. Ecosyst. Environ.* **2011**, *140*, 372–381, doi:10.1016/J.AGEE.2010.12.018.

4. Falloon, P.; Smith, P. Simulating SOC changes in long-term experiments with RothC and CENTURY: model evaluation for a regional scale application. *Soil Use Manag.* **2006**, *18*, 101–111, doi:10.1111/j.1475-2743.2002.tb00227.x.

5. Metropolis, N.; Ulam, S. The Monte Carlo method. *J. Am. Stat. Assoc.* **1949**, *44*, 335–341, doi:10.1080/01621459.1949.10483310.

6. IPCC *2006 IPCC Guidelines for National Greenhouse Gas Inventories. Institute for Global Environmental Strategies (IGES) for the Intergovernmental Panel on Climate Change.*; The Intergovernmental Panel on Climate Change (IPCC): Kanagawa, 2006;

7. IPCC *Revised 1996 IPCC Guidelines for National Greenhouse Gas Inventories. Volume 2 - Workbook*; The Intergovernmental Panel on Climate Change (IPCC), the Organization for Economic Co-operation and Development (OECD) and the International Energy Agency (IEA), 1997;

8. FAO Food and Agriculture Organization of the United Nations - Statistics Division Available online: http://faostat.fao.org/ (accessed on Mar 25, 2015).

9. IPCC *Good Practice Guidance for Land Use, Land-Use Change and Forestry. Institute for Global Environmental Strategies (IGES) for the Intergovernmental Panel on Climate Change*; The Intergovernmental Panel on Climate Change (IPCC): Kanagawa, 2003;

10. Mueller, N. D.; Gerber, J. S.; Johnston, M.; Ray, D. K.; Ramankutty, N.; Foley, J. A. Closing yield gaps through nutrient and water management. *Nature* **2012**, *490*, 254–257, doi:10.1038/nature11420.

11. Román, P.; Martínez, M. M.; Pantoja, A. Farmer’s Compost Handbook - Experiences in Latin America 2015.

12. Robinson, T. P.; William Wint, G. R.; Conchedda, G.; Van Boeckel, T. P.; Ercoli, V.; Palamara, E.; Cinardi, G.; D’Aietti, L.; Hay, S. I.; Gilbert, M. Mapping the global distribution of livestock. *PLoS One* **2014**, *9*, e96084, doi:10.1371/journal.pone.0096084.

13. DAAC, L. MODIS/Terra Land Surface Temperature and Emissivity Monthly L3 Global 0.05Deg CMG Available online: https://lpdaac.usgs.gov/dataset_discovery/modis/modis_products_table/mod11c3.

14. NASA Global Precipitation Analysis Available online: http://precip.gsfc.nasa.gov/.

15. Pfister, S.; Bayer, P.; Koehler, A.; Hellweg, S. Environmental impacts of water use in global crop production: hotspots and trade-offs with land use. *Environ. Sci. Technol.* **2011**, *45*, 5761–8, doi:10.1021/es1041755.

16. Chapagain, A. K.; Hoekstra, A. Y. *Water footprint of nations. Volume 1 : Main report*; 2004;

17. Thornthwaite, C. W. An Approach toward a Rational Classification of Climate. *Geogr. Rev.* **1948**, *38*, 55, doi:10.2307/210739.

18. FAO/IIASA Global Agro-ecological Zones (GAEZ v3.0). *IIASA, Laxenburg, Austria FAO, Rome, Italy. iv* **2012**, doi:10.1029/97GB03657.

19. NASA LP DAAC Land Cover Type Yearly L3 Global 0.05Deg CMG (MCD12C1) Available online: https://lpdaac.usgs.gov/dataset_discovery/modis/modis_products_table/mcd12c1 (accessed on Feb 20, 2017).

20. Fischer, G.; Nachtergaele, F.; Prieler, S.; van Velthuizen, H.; Verelst, L.; Wiberg, D. Global Agro-ecological Zones Assessment for Agriculture Available online: http://www.iiasa.ac.at/Research/LUC/luc07/External-World-soil-database/HTML/index.html?sb=1.

21. Thematicmapping.org World Borders Dataset Available online: http://thematicmapping.org/downloads/world_borders.php (accessed on Feb 20, 2017).

22. Coleman, K.; Jenkinson, D. *RothC, A model for the turnover of carbon in soil – Model description and users guide*; Rothamsted Research. Harpenden Herts, 2014;

23. Jebari, A. Estimación de los cambios en los stocks de carbono del suelo agrícola a escala regional: Impacto de los usos del suelo y del manejo en la Comunidad Autónoma de Aragón, Master Thesis, 2016.

24. ESDAC Global Soil Organic Carbon Estimates Available online: http://esdac.jrc.ec.europa.eu/content/global-soil-organic-carbon-estimates.

25. Weihermüller, L.; Graf, A.; Herbst, M.; Vereecken, H. Simple pedotransfer functions to initialize reactive carbon pools of the RothC model. *Eur. J. Soil Sci.* **2013**, *64*, 567–575, doi:10.1111/ejss.12036.

26. Tóth, G.; Jones, A.; Montanarella, L. The LUCAS topsoil database and derived information on the regional variability of cropland topsoil properties in the European Union. *Environ. Monit. Assess.* **2013**, *185*, 7409–25, doi:10.1007/s10661-013-3109-3.
