# Supplementary material for: Detailed global modelling of soil organic carbon in cropland, grassland and forest soils
Source: PLoS One. 2019 Sep 19;14(9):e0222604. doi: 10.1371/journal.pone.0222604 (PMC6752864; doi:10.1371/journal.pone.0222604)
Supplement: S3 File — (DOCX) [file pone.0222604.s003.docx]

Detailed global modelling of soil organic carbon in cropland, grassland and forest soils

Tiago G. Morais, Ricardo F.M. Teixeira and Tiago Domingos

**Supporting Information File S3**

**UHTU map**

Available at: <http://doi.org/10.5281/zenodo.3387665>.

File name: S3. UHTU map.docx.
