# Supplementary material for: Detailed global modelling of soil organic carbon in cropland, grassland and forest soils
Source: PLoS One. 2019 Sep 19;14(9):e0222604. doi: 10.1371/journal.pone.0222604 (PMC6752864; doi:10.1371/journal.pone.0222604)
Supplement: S4 File — (DOCX) [file pone.0222604.s004.docx]

Detailed global modelling of soil organic carbon in cropland, grassland and forest soils

Tiago G. Morais, Ricardo F.M. Teixeira and Tiago Domingos

**Supporting Information File S4**

**UHTU map**

Available at: <http://doi.org/10.5281/zenodo.3387665>.

File name: S4. Map results UHTU scale.xlsx.

This file contains:

| **Organic fertilization scenario 1 - No organic fertilizer application** | |
| --- | --- |
| Sheet: SOC_mean_no_fert | Average stabilization Soil Organic Carbon (SOC) stock (t C.ha-1) per unique homogeneous territorial units (UHTU) for all 79 land uses |
| Sheet: SOC_std_no_fert | Standard deviation stabilization Soil Organic Carbon (SOC) stock per unique homogeneous territorial units (UHTU) for all 79 land uses |
| Sheet: Min_rate_mean_no_fert | Average Mineralization Rate (%) per unique homogeneous territorial units (UHTU) for all 79 land uses |
| Sheet: Min_rate_std_no_fert | Standard deviation Mineralization Rate (%) per unique homogeneous territorial units (UHTU) for all 79 land uses |
| Sheet: K_mean_no_fert | Average K parameter (annual C input) per unique homogeneous territorial units (UHTU) for all 79 land uses |
| Sheet: K_std_no_fert | Standard deviation K parameter (annual C input) per unique homogeneous territorial units (UHTU) for all 79 land uses |
|  |  |
| **Organic fertilization scenario 2 - All of fertilizer application is organic** | |
| Sheet: SOC_mean_fert_100 | Average stabilization Soil Organic Carbon (SOC) stock (t C.ha-1) per unique homogeneous territorial units (UHTU) for all 79 land uses |
| Sheet: SOC_std_fert_100 | Standard deviation stabilization Soil Organic Carbon (SOC) stock per unique homogeneous territorial units (UHTU) for all 79 land uses |
| Sheet: Min_rate_mean_fert_100 | Average Mineralization Rate (%) per unique homogeneous territorial units (UHTU) for all 79 land uses |
| Sheet: Min_rate_std_fert_100 | Standard deviation Mineralization Rate (%) per unique homogeneous territorial units (UHTU) for all 79 land uses |
| Sheet: K_mean_fert_100 | Average K parameter (annual C input) per unique homogeneous territorial units (UHTU) for all 79 land uses |
| Sheet: K_std_fert_100 | Standard deviation K parameter (annual C input) per unique homogeneous territorial units (UHTU) for all 79 land uses |
|  |  |
| **Organic fertilization scenario 3 - 50% of fertilizer application is organic** | |
| Sheet: SOC_mean_fert_50 | Average stabilization Soil Organic Carbon (SOC) stock (t C.ha-1) per unique homogeneous territorial units (UHTU) for all 79 land uses |
| Sheet: SOC_std_fert_50 | Standard deviation stabilization Soil Organic Carbon (SOC) stock per unique homogeneous territorial units (UHTU) for all 79 land uses |
| Sheet: Min_rate_mean_fert_50 | Average Mineralization Rate (%) per unique homogeneous territorial units (UHTU) for all 79 land uses |
| Sheet: Min_rate_std_fert_50 | Standard deviation Mineralization Rate (%) per unique homogeneous territorial units (UHTU) for all 79 land uses |
| Sheet: K_mean_fert_50 | Average K parameter (annual C input) per unique homogeneous territorial units (UHTU) for all 79 land uses |
| Sheet: K_std_fert_50 | Standard deviation K parameter (annual C input) per unique homogeneous territorial units (UHTU) for all 79 land uses |
